# A Review of Multiscale Computational Methods in Polymeric Materials

**DOI:** 10.3390/polym9010016

**Published:** 2017-01-09

**Authors:** Ali Gooneie, Stephan Schuschnigg, Clemens Holzer

**Affiliations:** Chair of Polymer Processing, Montanuniversitaet Leoben, Otto Gloeckel-Strasse 2, 8700 Leoben, Austria; stephan.schuschnigg@unileoben.ac.at (S.S.); clemens.holzer@unileoben.ac.at (C.H.)

**Keywords:** computer simulations, computational methods, multiscale modelling, hierarchical structures, multiple scales, bridging strategies, polymers, nanocomposites

## Abstract

Polymeric materials display distinguished characteristics which stem from the interplay of phenomena at various length and time scales. Further development of polymer systems critically relies on a comprehensive understanding of the fundamentals of their hierarchical structure and behaviors. As such, the inherent multiscale nature of polymer systems is only reflected by a multiscale analysis which accounts for all important mechanisms. Since multiscale modelling is a rapidly growing multidisciplinary field, the emerging possibilities and challenges can be of a truly diverse nature. The present review attempts to provide a rather comprehensive overview of the recent developments in the field of multiscale modelling and simulation of polymeric materials. In order to understand the characteristics of the building blocks of multiscale methods, first a brief review of some significant computational methods at individual length and time scales is provided. These methods cover quantum mechanical scale, atomistic domain (Monte Carlo and molecular dynamics), mesoscopic scale (Brownian dynamics, dissipative particle dynamics, and lattice Boltzmann method), and finally macroscopic realm (finite element and volume methods). Afterwards, different prescriptions to envelope these methods in a multiscale strategy are discussed in details. Sequential, concurrent, and adaptive resolution schemes are presented along with the latest updates and ongoing challenges in research. In sequential methods, various systematic coarse-graining and backmapping approaches are addressed. For the concurrent strategy, we aimed to introduce the fundamentals and significant methods including the handshaking concept, energy-based, and force-based coupling approaches. Although such methods are very popular in metals and carbon nanomaterials, their use in polymeric materials is still limited. We have illustrated their applications in polymer science by several examples hoping for raising attention towards the existing possibilities. The relatively new adaptive resolution schemes are then covered including their advantages and shortcomings. Finally, some novel ideas in order to extend the reaches of atomistic techniques are reviewed. We conclude the review by outlining the existing challenges and possibilities for future research.

Contents
1. Introduction12. Simulation Methods5    2.1. Quantum Mechanics5    2.2. Atomistic Techniques6     2.2.1. Monte Carlo7     2.2.2. Molecular Dynamics8    2.3. Mesoscale Techniques9     2.3.1. Brownian Dynamics10     2.3.2. Dissipative Particle Dynamics11     2.3.3. Lattice Boltzmann12    2.4. Macroscale Techniques14     2.4.1. Finite Element Method15     2.4.2. Finite Volume Method173. Multiscale Strategies19    3.1. Sequential Multiscale Approaches19     3.1.1. Systematic Coarse-Graining Methods22     3.1.1.1. Low Coarse-Graining Degrees23     3.1.1.2. Medium Coarse-Graining Degrees26     3.1.1.3. High Coarse-Graining Degrees29     3.1.2. Reverse Mapping30    3.2. Concurrent Multiscale Approaches33     3.2.1. The Concept of Handshaking34     3.2.2. Linking Atomistic and Continuum Models35     3.2.2.1. Quasicontinuum Approach37     3.2.2.2. Coarse-Grained Molecular Dynamics39     3.2.2.3. Finite-element/Atomistic Method39     3.2.2.4. Bridging Scale Method40     3.2.2.5. Applications in Polymeric Materials41    3.3. Adaptive Resolution Simulations42     3.3.1. The Adaptive Resolution Scheme43     3.3.2. The Hamiltonian Adaptive Resolution Scheme45    3.4. Extending Atomistic Simulations474. Conclusions and Outlooks49Appendix A. Acronyms and Nomenclature51References56

## 1. Introduction

Polymeric materials display distinguished characteristics which range from the angstrom level of an individual bond, to tens of nanometers of the chain gyration radius, to micrometers, millimeters and larger in melts, blends, solutions and polymer nanocomposites (PNCs). The corresponding time scales of the dynamics relevant to different material properties span an even wider range from femtoseconds to seconds or even hours for large-scale ordering processes such as phase separation in blends. In order to highlight the inherent multiscale nature of polymer systems, two interesting cases from the literature are briefly outlined. Indeed, many other examples from various fields of polymer science can be found elsewhere [1,2,3,4,5,6,7,8,9,10,11,12,13]. We believe that the selected examples should suffice to serve the purpose as well as the brevity.

As the first example, PNCs are considered due to their importance to many applications. The incorporation of nanoparticles in polymers has attracted substantial academic and industrial interest due to the dramatic improvements in the properties of the host polymers. The addition of only 1–10 vol % nanoparticles has been shown to be able to enhance various properties of the neat polymers [14,15,16,17,18,19,20]. These changes are often introduced into the polymer matrix while many benefits of the neat polymer including rather easy processability are still preserved [21,22]. Therefore, PNCs are ideal candidates for multiple applications like medical devices, aerospace applications, automobile industries, coatings, etc. Experience has shown that the property enhancement in PNCs is directly linked to the nanoparticles arrangement and dispersion [21,23]. A precise morphology control is of great significance in PNCs, otherwise the full property potential of these materials cannot be achieved. The fact that many of the common nanoparticles possess strong van der Waals interactions promotes their aggregation and consequently diminishes their effectiveness. On the other hand, the role of polymer-particle interactions can either facilitate or complicate the aggregation process. Moreover, the geometrical characteristics of the nanoparticles, such as aspect ratio and structural flexibility, add to the complexity of their impact on the properties since it can alter surface energies as well as surface-to-volume ratio [24]. Therefore, the structural characterization and the detailed evaluation of the fabrication of PNCs are crucial to achieve the desired properties. Many studies are devoted to understand the effects of processing conditions on the final microstructure and the resulting properties of the PNCs [19,20,21,23,24,25,26,27]. The multiscale nature of PNCs simply divulges if one considers the interplaying role of the fabrication stage with macroscopic characteristics and the aforementioned submicron phenomena involved in the final outcome of PNCs.

A fascinating field of application for multiscale methods is in biological systems [3,4,7]. For instance, we take a single hair strand. It is well known that hairs, i.e., keratin fibers, exhibit a complex structure [28]. Filaments with a diameter of approximately 8 nm are tightly packed in a matrix, filling the approximately 2 nm gap in between which are later assembled into a so-called macrofibril. Often, several hundred filaments form one macrofibril. Various macrofibrils can be categorized based on how packed they are. These macrofibrils constitute the main part of the hair cells in the cortex. The remaining volume of the cell is comprised of the remnants and pigment granules. The cross-section of a hair typically has almost 100 cells, contained by a cell-membrane structure. Finally, the cortex is encapsulated by the cuticle which forms the surface of a hair fiber. It is of significance to be able to find the relation between the mechanical properties of these fibers and the structure of the keratin proteins, temperature, humidity and deformation rate. Obviously, such analysis necessitates a multiscale approach to capture the precise behavior of the hair mechanics as suggested by Akkermans and Warren [28].

In order to find appropriate solutions to these questions, several theories and computational methods were developed which could introduce new possibilities to design, predict and optimize the structures and properties of materials. At present, no single theory or computational method can cover various scales involved in polymeric materials. As a result, the bridging of length and time scales via a combination of various methods in a multiscale simulation framework is considered to be one of the most important topics in computational materials research. The resulting multiscale method is preferably supposed to predict macroscopic properties of polymeric materials from fundamental molecular processes. In order to build a multiscale simulation, often models and theories from four characteristics length and time scales are combined. They are roughly divided into the following scales.

**1. The quantum scale (~10^−10^ m, ~10^−12^ s):** The nuclei and electrons are the particles of interest at this scale and quantum mechanics (QM) methods are used to model their state. The possibility to study the phenomena associated with formation and rupture of chemical bonds, the changes in electrons configurations, and other similar phenomena are typical advantages of modelling at quantum scale.

**2. The atomistic scale (~10^−9^ m, ~10^−9^–10^−6^ s):** All atoms or small groups of atoms are explicitly represented and treated by single sites in atomistic simulations. The potential energy of the system is estimated using a number of different interactions which are collectively known as force fields. The typical interactions include the bonded and nonbonded interactions. The bonded interactions often consist of the bond length, the bond angle, and the bond dihedral potentials. The most typically used nonbonded interactions are Coulomb interactions and dispersion forces. Molecular dynamics (MD) and Monte Carlo (MC) simulation techniques are often used at this level to model atomic processes involving a larger group of atoms compared with QM.

**3. The mesoscopic scale (~10^−6^ m, ~10^−6^–10^−3^ s):** At mesoscopic scale, a molecule is usually described with a field or a microscopic particle generally known as a bead. In this way the molecular details are introduced implicitly which provides the opportunity to simulate the phenomena on longer length and time scales hardly accessible by atomistic methods. A good example for the field-based description of polymer systems is the Flory-Huggins model for the free energy of mixing in which the details of the system are summed up in model parameters. On the other hand, in particle-based models collections of particles are accumulated in beads through a coarse-graining procedure. The interactions between the beads are then used to characterize the system. Various methods have been developed to investigate the mesoscopic structures in polymeric systems including dissipative particle dynamics (DPD), Brownian dynamics (BD), lattice Boltzmann (LB), dynamic density functional theory (DDFT), and time-dependent Ginzburg-Landau (TDGL) theory.

**4. The macroscale (~10^−3^ m, ~1 s):** At this scale, the system is treated as a continuous medium and the discrete characteristics of atoms and molecules are ignored. The behavior of such a system is governed by constitutive laws which are often coupled with conservation laws to simulate various phenomena. All functions such as velocity and stress components are continuous except at a finite number of locations which separate continuity regions. The fundamental assumption at this scale is in replacing a heterogeneous material with an equivalent homogeneous model. The most important methods used to simulate systems at this scale are finite difference method (FDM), finite element method (FEM), and finite volume method (FVM).

Although several review papers are available on the topic of multiscale simulations in materials [1,2,3,4,5,6,7,8,9,10,11,12,29,30,31], a comprehensive discussion of its various aspects in polymer science is still needed. Some reports approach the objective by introducing different case studies and never actually detailing various categories of multiscale methods, while some others focus only on a specific topic in multiscale simulations such as coarse-graining or concurrent simulations. Here, we aim to provide an opportunity for the interested reader to explore how such techniques might be applied in their own area of specialty by focusing on the core concepts of major trends in this field all in one place. Consequently, we outline the basics of the methods and illustrate each one with a few examples from the vast field of polymeric systems. We organize the review as follows. In Section 2, we introduce some of the most significant computational methods used so far to model different scales. This part is not intended to provide detailed description of each method. Instead, we aim to emphasize different approaches, challenges, restrictions, and opportunities that models of each scale could generally possess. Since such models are the building blocks for the multiscale methods, it is important to note how they convey their characteristics into a multiscale approach. We strongly advice the interested reader to refer to relevant literature, some significant ones introduced here, for further information. In Section 3, we discuss in detail various ideas to link scales in a multiscale package. Four major blocks are presented in this part: Sequential Multiscale Approaches, Concurrent Multiscale Approaches, Adaptive Resolution Simulations, and Extending Atomistic Simulations. This section is the core of the paper and therefore we attempt to deliver the most recent advances in each instance. In every case, the applications in polymer science are highlighted to serve the topic. It was a serious concern of ours to cite the outstanding studies that could cover from the classic fundamental works up to the latest publications. We hope this eases further pursue of the relevant works. It should be noted that the topic at hand is massive and there might be some significant studies which are left out despite our attempts. Finally, we conclude the review by emphasizing the current challenges and future research directions. Overall, the present review is meant to put forth the major directions in multiscale simulation strategies in polymer science.

## 2. Simulation Methods

In general, computational methods are categorized into either particle-based or field-based approaches [32,33]. The particle-based methods incorporate particles to represent the building blocks of polymers such as atoms, molecules, monomers, or even an entire polymer chain. These particles (and their combinations in the form of bonds, angles, dihedrals and so on) often interact with each other through certain forces which form a force field altogether [34]. By the application of a statistical mechanical sampling method, the particles are allowed to move within a certain thermodynamic ensemble and hence simulate a desired process [35]. Perhaps the most well-known particle-based techniques are MD and its coarser versions such as DPD. In the second category, i.e., the field-based approaches, the system is typically described in terms of effective potentials, collective dynamic variables, and density fields which determine the degrees of freedom of the model [36]. Therefore, a reduced representation of the system is developed based on some phenomenological approximation [32]. The famous Flory approximation of the free energy of a polymer is a good example of the field-based strategy [37]. Another valuable field-based method is the polymer reference interaction site model (PRISM) which attempts to realize the polymer structure in terms of density correlation functions [38]. Other examples of such methods include density functional theory (DFT) [38,39,40], self-consistent field theory (SCFT) [32,33,38], and phase-field techniques [41,42,43]. In this section, we outline the details of some of the most important methods at different scales. These methods mainly belong to the particle-based approaches due to their relevance to the rest of the discussion as well as to our own research interest. For more details on the field-based methods, the reader is referred to the cited literature.

### 2.1. Quantum Mechanics

A precise treatment of atomistic scale phenomena requires the solution of the Schrödinger wave equations for all electrons and nuclei on the basis of a quantum scale modelling [44]. In QM, the time-independent form of the wave equation ${\varphi (r)}_{k}$ for a particle in an energy eigenstate $E_{k}$ in a potential U(r) having coordinates vector r and mass m is

(1) $$-\frac{h^{2}}{{8\pi }^{2}m}\nabla^{2}{\varphi (r)}_{k}+U(r){\varphi (r)}_{k}=E_{k}{\varphi (r)}_{k},$$

where h is Planck’s constant. It can be shown that for a material having i electrons with mass $m_{el}$ and the negative unit charge of −𝕖 and the coordinates $r_{{el}_{i}}$, and j nuclei with mass $m_{n}$ and a positive unit charge of $z_{n}𝕖$ with $z_{n}$ being the atomic number, and the spatial coordinates $r_{n_{j}}$, Equation (1) becomes

(2) $$-\frac{h^{2}}{{8\pi }^{2}m_{el}}\underset{i}{\sum }\nabla_{i}^{2}{\varphi (r}_{{el}_{1}}{,r}_{{el}_{2}}{,\ldots ,r}_{{el}_{i}}{,r}_{n_{1}}{,r}_{n_{2}}{,\ldots ,r}_{n_{j}}{)}_{k}\;-\frac{h^{2}}{{8\pi }^{2}}\underset{j}{\sum }\frac{1}{m_{n_{j}}}\nabla_{j}^{2}{\varphi (r}_{{el}_{1}}{,r}_{{el}_{2}}{,\ldots ,r}_{{el}_{i}}{,r}_{n_{1}}{,r}_{n_{2}}{,\ldots ,r}_{n_{j}}{)}_{k}\;+(\underset{i_{1}\neq i_{2}}{\underset{i_{1},i_{2}}{\sum }}\frac{{𝕖}^{2}}{\left|r_{{el}_{i1}}-r_{{el}_{i2}}\right|}+\underset{i,j}{\sum }\frac{z_{j}{𝕖}^{2}}{\left|r_{{el}_{i}}-r_{n_{j}}\right|}+\underset{j_{1}\neq j_{2}}{\underset{j_{1},j_{2}}{\sum }}\frac{z_{j1}z_{j2}{𝕖}^{2}}{\left|r_{n_{j1}}-r_{n_{j2}}\right|}){\varphi (r}_{{el}_{1}}{,r}_{{el}_{2}}{,\ldots ,r}_{{el}_{i}}{,r}_{n_{1}}{,r}_{n_{2}}{,\ldots ,r}_{n_{j}}{)}_{k}\;{=\;E}_{k}{\varphi (r}_{{el}_{1}}{,r}_{{el}_{2}}{,\ldots ,r}_{{el}_{i}}{,r}_{n_{1}}{,r}_{n_{2}}{,\ldots ,r}_{n_{j}}{)}_{k}\;.$$

In 1927, Born and Oppenheimer [45] proposed a strategy to separate the wave functions of the light electrons from the heavy nuclei considering that the electrons typically relax to some orders of magnitude faster than the nuclei. This strategy, known as the adiabatic Born-Oppenheimer approximation, assumes that the electrons always remain in their ground state irrespective of the positions of the nuclei by adiabatically adjusting to the movements of the nuclei. As a result of this assumption, one can define the wave function φ in Equation (2) as the product of two independent wave functions. In this approach, one function describes the dynamics of the electrons ϖ and the other function describes the dynamics of the nuclei ϕ. This can be shown as

(3) $${\varphi (r}_{{el}_{1}}{,r}_{{el}_{2}}{,\ldots ,r}_{{el}_{i}}{,r}_{n_{1}}{,r}_{n_{2}}{,\ldots ,r}_{n_{j}})\;=\;\varpi {(r}_{{el}_{1}}{,r}_{{el}_{2}}{,\ldots ,r}_{{el}_{i}})\;\phi {(r}_{n_{1}}{,r}_{n_{2}}{,\ldots ,r}_{n_{j}}).$$

Consequently, the corresponding wave function of the electrons with the eigenstate energy $E_{k_{el}}$ is

(4) $$\left(-\frac{h^{2}}{{8\pi }^{2}m_{el}}\underset{i}{\sum }\nabla_{i}^{2}+\underset{\begin{array}{c} i1,i2\\ i1\neq i2 \end{array}}{\sum }\frac{{𝕖}^{2}}{\left|r_{{el}_{i1}}{-r}_{{el}_{i2}}\right|}+\underset{i,j}{\sum }\frac{z_{j}{𝕖}^{2}}{\left|r_{{el}_{i}}{-r}_{n_{j}}\right|}\right)\varpi {{(r}_{{el}_{1}}{,r}_{{el}_{2}}{,\ldots ,r}_{{el}_{i}})}_{k_{el}}=\;E_{k_{el}}^{el}\varpi {{(r}_{{el}_{1}}{,r}_{{el}_{2}}{,\ldots ,r}_{{el}_{i}})}_{k_{el}},$$

and the corresponding wave function of the nuclei with the eigenstate energy $E_{k_{n}}$ is

(5) $$\left(-\frac{h^{2}}{{8\pi }^{2}}\sum_{j}\frac{1}{m_{n}}\nabla_{j}^{2}\;+\;\sum_{i,j}\frac{z_{j}{𝕖}^{2}}{\left|r_{{el}_{i}}{-r}_{n_{j}}\right|}\;+\;\sum_{\begin{array}{c} j1,j2\\ j1\neq j2 \end{array}}\frac{z_{j1}z_{j2}{𝕖}^{2}}{\left|r_{n_{j1}}{-r}_{n_{j2}}\right|}\right)\phi {{(r}_{n_{1}}{,r}_{n_{2}}{,\ldots ,r}_{n_{j}})}_{k_{n}}{=\;E}_{k_{n}}^{n}\phi {{(r}_{n_{1}}{,r}_{n_{2}}{,\ldots ,r}_{n_{j}})}_{k_{n}}.$$

It is worthy to note at this point that the use of the adiabatic Born-Oppenheimer approximation is justified only when the energy gap between ground and excited electronic states is larger than the energy scale of the nucleus motion. This assumption has been shown to fail in materials with zero energy gaps such as metals [46,47] and the free-state graphene [48]. Despite this, the adiabatic Born-Oppenheimer approximation has proved effective in the atomistic simulations of some metallic [49] and graphene-based systems [50] as well.

The quantum mechanical many-body problem was formulated by Kohn and Sham [40] in the density functional theory (DFT). In DFT, electrons were replaced by effective electrons with the same total density moving in the potential generated by the other electrons and ion cores. Later, DFT was modified by Car and Parrinello [51] which allowed for the movements to be incorporated into the DFT scheme, thus leading to the so-called ab initio MD (AIMD). Such methods have found useful applications in polymer science such as the simulation of mechanics of polyethylene (PE) macromolecules [52,53,54], conduction in polymers [55,56,57], polymerization [58,59], crystal structures [60], disordered conformations of poly(tetra fluoro ethylene) chains [61], and diffusion in polymers [62].

### 2.2. Atomistic Techniques

Atomistic scale simulations often benefit from Equation (5) to predict the initial atomic configurations assuming that the electrons are instantaneously equilibrated during the movements of the nuclei. The approximation methods of this equation are mainly divided into stochastic and deterministic approaches. The stochastic approaches are often referred to as MC methods which are well-credited to evaluate equilibrium states for certain distribution functions or to solve the equations of motion in their corresponding integral form. The deterministic approaches are typically referred to as MD which are mainly used to discretely solve the equation of motion. In general, simulations at this scale provide an atomistic picture of the interactions between components and conformational dynamics which could help uncover the underlying phenomena. By the way of illustration, we consider an example of the application of MD to PNCs in the work of Piscitelli et al. [63] who investigated the functionalization of sodium montmorillonite (Na-MMT) using three aminosilanes characterized by different lengths of the alkyl chains. It is known that the presence of negative charges on the surface of each MMT layer as well as counteracting cations such as sodium or potassium located in the vicinity of the platelets within the galleries produce highly polar pristine structures of Na-MMT [14,21,23]. These structures further lead to their incompatibility with the majority of polymers. Consequently, a simple dispersion of Na-MMT in a polymer results in the formation of aggregated structures within the matrix which is followed by the deterioration of the property enhancement in these PNCs. In order to avoid these structures, chemical functionalization of Na-MMT platelets like silylation reaction is often performed [14]. The X-ray diffraction (XRD) patterns of Piscitelli et al. [63] indicated that the silylation reaction results in the Na-MMT galleries to open up regardless of the type of the aminosilane. However, it was observed that the *d*-spacing in the modified Na-MMT was reduced as the organic chain of the aminosilane molecule became longer. This outcome might not be expected before the experiments and therefore MD was incorporated to illuminate the underlying phenomena. The simulations revealed the increasing tendency of aminosilane molecules with increasing their length to interact among themselves by intermolecular hydrogen bonding as well as hydrophobic interactions. These interactions could eventually lead to the bridging of aminosilane molecules between two Na-MMT layers for longer chains. This situation not only does not improve the *d*-spacing of the modified Na-MMT compared with the unmodified nanoparticles, but also acts against any attempts from polymer macromolecules to open up the layers. As observed in these simulations, MD can play a key role in the understanding of molecular mechanisms involved in the intercalation process in polymer/clay nanocomposites. Without a thorough vision of such molecular processes in aminosilane-functionalized Na-MMTs, the designed PNC would fail due to this general belief that longer organic chains normally result in higher interlayer spacing. In the following, MC and MD techniques are revisited.

#### 2.2.1. Monte Carlo

In general, the MC methods include a large number of stochastic computer experiments by incorporating uncorrelated random numbers. MC can be used to mimic stationary ensembles by exploring a multitude of states in the corresponding phase space. Therefore, one can obtain pseudo-time-averaged statistical data by calculating ensemble averages along trajectories in the phase space assuming the ergodic system behavior [64,65,66]. It should be noted that the MC methods are not restricted to the atomistic scale but can be used at any scale if an appropriate probabilistic model is provided.

MC methods often consist of three characteristic steps. These steps are: (i) translation of the physical phenomena under investigation into an analogous probabilistic or statistical model; (ii) solving the resulting probabilistic model by a large number of numerical stochastic sampling experiments; and (iii) analyzing the generated data utilizing statistical methods. The sampling method can follow either a simple sampling algorithm or a weighted sampling algorithm. The simple sampling uses an equal distribution of the random numbers while the weighted sampling develops random numbers based on a distribution which is accommodated to the problem being investigated. The weighted sampling algorithm is the underlying principle of the so-called Metropolis MC algorithm [67].

In Metropolis MC for canonical and/or microcanonical ensembles with N atoms, a new configuration of the atoms is achieved by randomly or systematically choosing one atom and moving it from its initial position i to the temporary trial position j. Consequently, the initial state $\Gamma_{i}$ of the system in the corresponding phase space is changed to the trial state $\Gamma_{j}$. This displacement alters the Hamiltonian of the system from ${H(\Gamma }_{i})$ to ${H(\Gamma }_{j})$ according to the particular interactions being considered in the model. Therefore, the change in the system Hamiltonian ${\Delta H(\Gamma }_{i\to j})$ is

(6) $${\Delta H(\Gamma }_{i\to j})\;=\;H(\Gamma_{j})\;-\;H(\Gamma_{i}).$$

If the imposed movement of the chosen atom brings the system to a lower state of energy, i.e., ${\Delta H(\Gamma }_{i\to j})\;<0$, the movement is accepted and the displaced atom remains in its new position. Otherwise, the imposed movement is only accepted with a certain probability $p_{i\to j}$ which is proportional to

(7) $$p_{i\to j}\;\propto \;\exp\left(-\frac{{\Delta H(\Gamma }_{i\to j})}{k_{B}T}\right),$$

where $k_{B}$ is Boltzmann’s constant, and T is temperature. In Metropolis MC, a random number ζ between 0 and 1 is generated and used to test the new configuration. The imposed movement is accepted only if $\zeta \;\leq \;\exp\left(-\frac{{\Delta H(\Gamma }_{i\to j})}{k_{B}T}\right)$. If the movement is not accepted, the initial position is assumed to be the new position and the entire procedure is repeated by considering another randomly chosen atom.

The Metropolis MC also suggests using the same strategy for the grandcanonical ensemble where the number of initial atoms might change. For this purpose, the change in the system energy due to the exchange of an arbitrarily chosen atom by an atom of a different kind is taken into account to determine whether the new configuration is accepted or not. The methodology is the same as before.

As a final remark on MC, it should be noted that the original MC methods were intrinsically designed to simulate the equilibrium states of a system. The extension of the MC predictions to the simulation of microstructure evolution was first promoted by the incorporation of Ising lattice model in Potts-type MC models [68,69,70]. In the sense of using an internal kinetic measure such as the number of MC steps, this class of MC models is often referred to as kinetic MC models [71,72,73,74,75].

MC simulations have been utilized to describe a variety of phenomena in polymeric materials. Its application covers a wide range of problems including study of polymer degradation [71,73], development of surface morphology in thin films [76,77,78,79,80], heterophase interfaces [81,82,83,84,85,86,87,88,89,90,91,92,93,94], crystal growth and melting [95,96,97,98], morphology evolution [99,100,101,102,103,104,105,106], fracture behavior [107], diffusion [108,109,110,111], study of polymer melt viscoelasticity by nonequilibrium MC [112,113], and prediction of phase diagrams [114,115].

#### 2.2.2. Molecular Dynamics

The MD method is a deterministic simulation technique for the simulation of many-body interaction phenomena at the atomistic scale. It is based on substituting the quantum mechanical expression for the kinetic energy in Equation (5) by the classic momentum term and solving it for a nucleon using Newton’s law of motion. Consequently, the simulation of a many-body system would require the formulation and solution of equations of motion of all constituting particles. The equation of motion of a particle i is

(8) $$m_{i}\frac{d^{2}r_{i}}{{\mathrm{dt}}^{2}}{=\;f}_{i},$$

where $m_{i}$ is the particle mass and $r_{i}$ is the particle position vector. $f_{i}$ is the force acting on the ith particle at time t which is obtained as the negative gradient of the interaction potential U, i.e., $f_{i}\;=\;-\nabla U\;=\;-(\frac{\partial U}{\partial x}i\;+\;\frac{\partial U}{\partial y}j\;+\;\frac{\partial U}{\partial z}k)$. The underlying potentials are often quantified in terms of the relative position of two or more particles. This means that these potentials together with their parameters, i.e., the so-called force field, describe how the potential energy of a many-body system depends on the coordinates of the particles [34,116]. Such a force field can be obtained by QM, empirical methods, and quantum-empirical methods. It should be noted that the criteria for selecting an adequate force field should address the necessary precision in the system description, transferability, and computational speed.

The overall algorithm of MD is to simulate the evolution of particle configurations based on an adequate force field by integrating the equations of motion over discrete steps in time. The procedure is simply to calculate the position and velocity of every particle at present and a time step later. The system of equations of motion of N particles can be solved by utilizing FDM. The Verlet technique is possibly the most common integration scheme among all [117,118]. Utilizing the Taylor expansion, it uses the positions $r_{i}\left(t\right)$ and accelerations $a_{i}\left(t\right)$ at time t, and positions $r_{i}\left(t\;-\;∆t\right)$ from the previous time step t − ∆t, to calculate the new positions $r_{i}\left(t\;+\;∆t\right)$ at the next time t + ∆t according to

(9) $$r_{i}\left(t\;+\;∆t\right)\;\approx {\;2r}_{i}\left(t\right)\;{-\;r}_{i}\left(t-∆t\right){\;+\;a}_{i}\left(t\right){\left(∆t\right)}^{2}.$$

The velocities $v_{i}\left(t\right)$ and $v_{i}\left(t\;+\;\frac{1}{2}∆t\;\right)$ at times t and $t\;+\;\frac{1}{2}∆t$ can be estimated as

(10) $$v_{i}\left(t\right)\;\approx \;\frac{r_{i}\left(t\;+\;∆t\right){\;-\;r}_{i}\left(t\;-\;∆t\right)}{2∆t},$$

(11) $$v_{i}\left(t\;+\;\frac{1}{2}∆t\;\right)\;\approx \;\frac{r_{i}\left(t\;+\;∆t\right){\;-\;r}_{i}\left(t\right)}{∆t}.$$

A typical interaction potential U may consist of a number of bonded and nonbonded interaction terms. The bonded interactions may include bond stretching, bond angle bending, dihedral angle torsion, and inversion interaction potentials described by various functions such as harmonic functions. The nonbonded interactions contain electrostatic and van der Waals contributions and may consist of various potential types such as Lennard-Jones potential, Buckingham potential, Coulombic potential, etc. The concept of using interaction potentials makes it possible to carry out atomistic MD simulations which reveal the atomistic mechanisms and intrinsic structural properties by considering a relatively large number of particles.

While MD is shown to be a promising and reliable method in atomistic scale modelling, it has statistical limitations. A comparison of MC and MD methods suggests that in a phase space with 6N degrees of freedom, N being the total number of particles, MC allows one to investigate many more states than MD. Therefore, the validity of ensemble averages obtained by MD is limited to the assumption of system ergodicity; an assumption which is not unambiguously proven [64]. Still, the great power of MD is its proficiency to predict microstructure dynamics along its deterministic trajectory at an atomistic level. Applications of MD in the field of polymeric materials include topics such as macromolecular dynamics [119,120,121,122,123,124], intercalation phenomena in polymer/clay nanocomposites [63], structure of interfaces [125,126,127], polymer membranes [128,129], crystal structures [130,131,132], diffusion phenomena [133,134,135,136], segregation phenomena [137], tribological properties and crack propagation [138,139,140], thin films and surfaces [141,142,143,144], liquid crystalline polymers [145,146], rheology of polymeric systems [147,148,149,150], application of elongational flows on polymers using nonequilibrium MD [151,152], and the simulations of reactive systems such as crosslinking and decomposition of polymers using the ReaxFF force field [153,154,155,156].

### 2.3. Mesoscale Techniques

Atomistic simulations of complex systems including polymeric materials provide a detailed picture of, for instance, the interactions between components and conformational dynamics. Such information is often missing in macroscale models. On the other hand, the description of hydrodynamic behavior is relatively straightforward to handle in macroscale methods while it is challenging and expensive to address in atomistic models. Between the domains of these scale ranges, there is the intermediate mesoscopic scale which extends the time scale of atomistic methods. To show the importance of the time scale in the observed phenomena in soft matters, we take the lipid bilayers as an example. Bonds and angles of lipid molecules fluctuate within a time scale of a few picoseconds [157]. If the time scale is increased by an order of magnitude, trans-gauche isomerizations of dihedrals take place [158]. By further increasing the time scale to a few nanoseconds, the phospholipid molecule rotates around its axis. Moving on to longer time scales, two lipids can switch places in a bilayer on a time scale of tens of nanoseconds. Moreover, the individual lipid molecules orient and form membranes protrusions [159]. The peristaltic motions and undulations take place on a scale of 100 ns [160]. Finally, the steady transverse diffusion of lipids dominates on a time scale of 2 ms [161]. Simulating such a wide range of time scales in a single atomistic MD model needs large-scale computational resources. Consequently, the various mesoscale methods are developed which attempt to link atomistic and macroscale techniques and compensate for their shortcomings. Here, we briefly review BD, DPD and LB techniques which are often used at this scale. In addition to these methods, we also refer the interested reader to the stochastic multiparticle collision model developed by Malevanets and Kapral [162] to investigate complex fluids such as polymers. This method was recently coupled with MD and an adaptive resolution hybrid model was achieved which is particularly interesting to study transport and hydrodynamic properties [163].

#### 2.3.1. Brownian Dynamics

The motions of colloidal particles in dilute dispersions are a common example to introduce the BD method. Since the solvent molecules are often much smaller than the colloidal particles, the characteristic time of the motions of the solvent molecules is much smaller than that of the particles. Therefore, if one observes such dispersions based on the characteristic time of the solvent molecules in a MD framework, the suspended particles seem quiescent. In this case, a very long simulation time is necessary in order to observe the motions of particles. Hence, performing MD simulations is unrealistic when it is necessary, for instance, to trace a particle in time in order to calculate the diffusion coefficient. BD method overcomes this difficulty by replacing the explicit solvent molecules in MD with an implicit continuum medium. In BD simulations, the effects of the solvent molecules on the colloidal particles are defined by dissipative and random forces.

If the dispersion is dilute enough to neglect the hydrodynamic interactions between particles, the Brownian motion of particle i is generally described by the Langevin equation as [164]

(12) $$m_{i}\frac{d^{2}r_{i}}{{\mathrm{dt}}^{2}}{=\;f}_{i}{\;-\;\xi v}_{i}{\;+\;f}_{i}^{B}.$$

In this equation, $m_{i}$, $r_{i}$ and $v_{i}$ are the mass, position and velocity vectors of the particle i, $f_{i}$ is the sum of the forces exerted on particle i by the other particles, and ξ is the friction coefficient. Here, $f_{i}^{B}$ is the random force inducing the Brownian motions of the particle due to the motions of solvent molecules. The random force should be independent of the particle position and velocity and is described by its stochastic properties

(13) $$\langle f_{i}^{B}(t)\rangle \;=\;0,$$

(14) $$\left\langle f_{i}^{B}(t)\;\cdot \;f_{i}^{B}(t\prime )\rangle \;=\;A\delta (t-t\prime \right),$$

where δ(t−t′) is the Dirac delta function and ${A\;=\;6\xi k}_{B}T$. The position and velocity of each particle in time is therefore described as

(15) $$r_{i}\left(t\;+\;∆t\right){\;=\;r}_{i}\left(t\right)\;+\;\frac{m_{i}}{\xi }v_{i}\left(t\right)\left({1\;-\;e}^{-\;\frac{\xi }{m_{i}}\Delta t}\right)\;+\;\frac{1}{\xi }f_{i}\left(t\right)\left(\Delta t\;-\;\frac{m_{i}}{\xi }\left({1\;-\;e}^{-\;\frac{\xi }{m_{i}}\Delta t}\right)\right){\;+\;\delta r}_{i}^{B}(t\;+\;\Delta t),$$

(16) $$v_{i}\left(t\;+\;∆t\right){\;=\;v}_{i}\left(t\right)e^{-\;\frac{\xi }{m_{i}}\Delta t}\;+\;\frac{1}{\xi }f_{i}\left(t\right)\left({1\;-\;e}^{-\;\frac{\xi }{m_{i}}\Delta t}\right){\;+\;\delta v}_{i}^{B}(t\;+\;\Delta t).$$

The terms ${\delta r}_{i}^{B}(t\;+\;\Delta t)$ and ${\delta v}_{i}^{B}(t\;+\;\Delta t)$ represent a random displacement and velocity change due to the random forces. One can utilize a two-dimensional normal distribution to sample these terms based on random numbers [165]. Consequently, the positions and velocities of the particles can be updated in every time step during the simulations. It should be noted that the momentum is not conserved in the formulation of BD due to the random noise terms. As a result, BD cannot reproduce correct hydrodynamics and is limited to the prediction of diffusion properties [164,166,167].

If the dispersion is not dilute and the hydrodynamic interactions between the particles are not negligible, the above equations should be modified. Ermak and McCammon [168] have introduced such effects into BD. In their method, the diffusion tensor is utilized to re-write the Langevin equation. Recently, Ando et al. [169] suggested to use Krylov subspaces for computing Brownian random noise vectors. Their method facilitates performing large-scale BD simulations with hydrodynamic interactions. They showed that only low accuracy is required in the Brownian noise vectors to accurately evaluate dynamic and static properties of model polymer and monodisperse suspensions. BD has been incorporated to study a variety of phenomena including particle dispersions [170,171,172,173,174,175,176,177], polymer solutions [178,179,180,181], confined suspensions [182], peeling behavior of polymer molecules from a surface [183], and translocation of complex molecules through nanopores [184,185].

#### 2.3.2. Dissipative Particle Dynamics

DPD is a relatively new mesoscopic particle simulation method proposed by Hoogerbrugge and Koelman in 1992 [186]. Fundamentally, DPD is similar to MD except for the fact that individual DPD particles (which are often referred to as beads in the literature) represent the dynamic behavior of several atoms or molecules. This coarse-graining strategy along with the softer potential functions incorporated to represent bead-bead interactions allow for the simulation of dynamic processes over longer time scales [187,188].

In DPD, the motion of each bead is dominated by three pairwise forces. For bead i with the mass $m_{i}$ and position vector $r_{i}$, the Newton’s equation of motion becomes

(17) $$m_{i}\frac{d^{2}r_{i}}{{\mathrm{dt}}^{2}}\;=\;\sum_{j}\left(F_{ij}^{C}{\;+\;F}_{ij}^{D}{\;+\;F}_{ij}^{R}\right),$$

in which $F_{ij}^{C}$, $F_{ij}^{D}$, and $F_{ij}^{R}$ are respectively the conservative, the dissipative, and the random forces between bead i and its neighboring beads within a certain force cutoff radius $r_{\mathrm{cut}}$. These forces are defined as [187]

(18) $$F_{ij}^{C}=A_{ij}\chi_{ij}(1-\frac{r_{ij}}{r_{cut}}){\hat{r}}_{ij},$$

(19) $$F_{ij}^{D}=-\xi_{ij}\omega^{D}(r_{ij})r_{ij}[(v_{i}-v_{j})\cdot {\hat{r}}_{ij}]{\hat{r}}_{ij},$$

(20) $$F_{ij}^{R}=\sigma_{ij}\omega^{R}(r_{ij})r_{ij}\zeta_{ij}{\hat{r}}_{ij}.$$

Here, $r_{ij}$ is the distance between the beads i and j, is the unit vector pointing from the center of bead j to that of bead i, $\chi_{ij}$ equals 1 for beads with a distance less than $r_{\mathrm{cut}}$ and equals 0 otherwise. $v_{i}$ and $v_{j}$ are the velocity vectors of the ith and jth beads, respectively. $\zeta_{ij}$ is a Gaussian random number with zero mean and unit variance. $A_{ij}$ is the maximum repulsion between bead i and bead j. $\xi_{ij}$ and $\sigma_{ij}$ are the friction coefficient and the noise amplitude between bead i and bead j, respectively. $\omega^{D}\left(r_{ij}\right)$ and $\omega^{R}\left(r_{ij}\right)$ are dissipative and random weight functions, respectively. DPD simulations often obey the fluctuation-dissipation theorem in which one of the two weight functions fixes the other one [189]. This theory dictates that the random and dissipative terms must be administered in a particular way in order to maintain the correct Boltzmann distribution in equilibrium. As a consequent of this theory, one has

(21) $$\omega^{D}\left(r_{ij}\right)\;=\;{\left[\omega^{R}\left(r_{ij}\right)\right]}^{2},$$

(22) $${\sigma_{ij}}^{2}{\;=\;2\xi }_{ij}k_{B}T.$$

These relationships ensure an equilibrium distribution of bead velocities for thermodynamic equilibrium. In many studies, the weight functions are

(23) $$\omega^{D}\left(r_{ij}\right)\;=\;{\left[\omega^{R}\left(r_{ij}\right)\right]}^{2}{\;=\;\chi }_{ij}{\left(1\;-\;\frac{r_{ij}}{r_{cut}}\right)}^{2}.$$

Due to the pairwise nature of the forces involved in DPD framework, all of the beads obey Newton’s third law [190]. As a result, the sum of all forces in the system vanishes. Furthermore, any given volume of beads in the system is only accelerated by the sum of all forces that cross its boundaries. This is the fundamental assumption which results in the Navier-Stokes equation. Consequently, DPD formulation conserves hydrodynamics [187,190,191]. If the random force was not pairwise as in BD formulation see Equation (12), momentum would not be conserved [164,165].

At every time step during the simulation, the set of positions and velocities of the beads is updated utilizing the positions and velocities at the earlier time. In principle, all algebraic update algorithms from MD can be used in DPD. However, the dependence of forces on velocity in DPD complicates the algorithm. A common approach to solve this problem is to use a modified version of the velocity-Verlet algorithm [117,118,187]. For bead i with unit mass and the overall force $f_{i}$ over a short interval of time ∆t, the algorithm suggests

(24) $$r_{i}\left(t\;+\;∆t\right)\;\approx {\;r}_{i}\left(t\right){\;+\;v}_{i}\left(t\right)\;∆t\;+\;\frac{1}{2}f_{i}\left(t\right){\left(∆t\right)}^{2},$$

(25) $${\tilde{v}}_{i}\left(t\;+\;∆t\right)\;\approx {\;v}_{i}\left(t\right){\;+\;\lambda \;f}_{i}\left(t\right)\;∆t,$$

(26) $$f_{i}\left(t\;+\;∆t\right)\;\approx {\;f}_{i}\left(r_{i}(t\;+\;∆t),{\tilde{v}}_{i}\left(t\;+\;∆t\right)\right),$$

(27) $$v_{i}\left(t\;+\;∆t\right)\;\approx {\;v}_{i}\left(t\right)\;+\;\frac{1}{2}∆{t\;(f}_{i}\left(t\right){\;+\;f}_{i}\left(t\;+\;∆t\right)).$$

In this algorithm, the velocity in the next time step is first estimated by a predictor method, i.e., ${\tilde{v}}_{i}\left(t\;+\;∆t\right)$ and then corrected in the last step, i.e., $v_{i}\left(t\;+\;∆t\right)$. If the forces were independent of velocity, the actual velocity-Verlet algorithm would be recovered for λ = 0.5. The parameter λ has been shown to affect the temperature in DPD simulations by Den Otter and Clarke [192]. Based on empirical observations, some authors suggest λ = 0.65 would yield an accurate temperature control probably due to the cancellation of errors [190].

In recent years, modified versions of DPD formulation have been developed. For instance, Pan et al. [193] formulated DPD by borrowing ideas from fluid particle model. This approach enabled an explicit separation of dissipative forces into central and shear components. As a further consequence of this methodology, the hydrodynamics of Brownian colloidal suspensions were correctly captured by redistributing and balancing the forces. In another study, Yamanoi et al. [194] replaced the conservative forces with entanglement forces in the force field to reproduce the physics of entangled polymers. In this way, they could successfully simulate static as well as dynamic behavior of linear polymer melts. Despite these efforts, the standard DPD has also shown quite capable of simulating complex systems such as compatibilized and uncompatibilized polymer/clay nanocomposites under shear flows [195,196]. Various polymeric systems have been successfully treated in the DPD framework such as blood rheology [197,198,199], rheology of ultrahigh molecular weight polymers [200], lipid bilayers [161], adsorption characteristics of confined PE glycols dissolved in water [201], crosslinking of thermoset resins and formation of a network in the bulk [202], structure of thermoset polymers near an alumina substrate [203], graphene structure [204], surfactant aggregation [205], photo degradation process of polymer coatings [71], distribution of nanoparticles in lamellar and hexagonal diblock copolymer matrices [206,207], surface segregation and self-repairing systems [208,209,210], and electrical percolation threshold in packed assemblies of oriented fiber suspensions [211].

#### 2.3.3. Lattice Boltzmann

While BD and DPD techniques borrow ideas from MD to tackle the challenges at the mesoscale, some other methods such as lattice gas cellular automata (LGCA) and LB incorporate kinetic theory concepts. In this part of the paper, we briefly point out the fundamental ideas of LGCA at first and afterwards introduce LB as a pre-averaged version of LGCA.

LGCA was initially designed to overcome the computational limitations in the study of fluids at high Reynolds numbers (Re) [212]. In this method, the particles of fluid are bound to move on the nodes of a discrete lattice at discrete time steps. At each time step particles can move from one lattice node to a neighboring node according to a set of prescribed velocity vectors $\left\{{𝕧}_{k}\right\}$ which connect the neighboring nodes. In addition, only single occupancy is allowed for each possible velocity at a given node. The dynamics has two steps according to LGCA: (i) a propagation step, and (ii) a collision step. In the propagation step, also known as the streaming step, the particles move from their current node to an empty neighboring node with respect to their velocity. In the collision step, the particles collide and scatter according to certain rules which honor the mass and momentum conservation. In this way the Navier-Stokes equations are simulated correctly provided that the lattice and the velocity space are chosen carefully [164,165]. Although LGCA is unconditionally stable, it does not allow as large Re as it was initially thought [166].

LB inherits the discretized lattice dynamics based on propagation and collision steps from LGCA. However, it incorporates a one-particle distribution function as the relevant dynamic variable instead of the particle-based dynamics in LGCA. Initially, the collisions in LB is modelled by pre-averaging the collision schemes in the underlying LGCA model [213]. The resulting collision mechanism is then presented by a linearized collision matrix in which the distribution function relaxes toward a local equilibrium distribution [214,215]. In the LB scheme, thermal noises are not present which makes it much more efficient in comparison with LGCA for hydrodynamic problems. On the other hand, the intrinsic stability of LGCA is lost in LB. It should be noted that both LGCA and LB methods suffer from Galilean invariance problems and should be corrected for these limitations [166].

**Figure 1:**
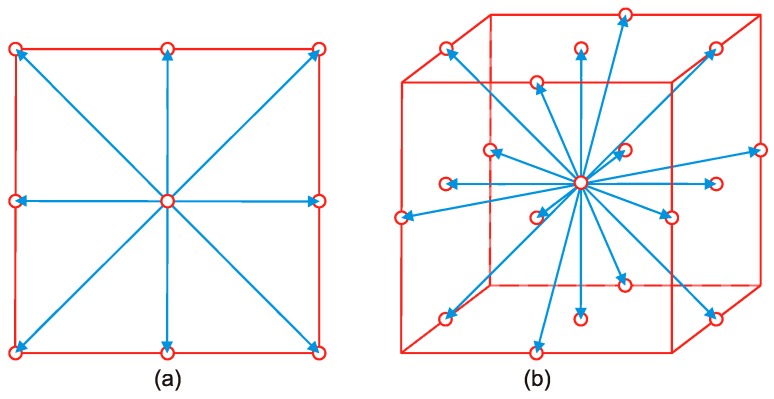
Two typical lattices often used in LB simulations: (**a**) D2Q9; and (**b**) D3Q19.

The particle distribution function $\Psi_{i}(r,t)$ used in LB gives the density of particles at node r at time t moving with velocity ${𝕧}_{i}$ in the i-direction. The lattice in which this density moves is characterized by both the sets of constructing nodes and the velocity subspace $\left\{{𝕧}_{k}\right\}$. The velocity subspace determines the neighboring nodes to which a given density will be able to move in a time step. The lattice symmetry and the minimum allowed set of velocities should satisfy the requirement of a minimum set of symmetry properties. Otherwise, the underlying anisotropy of the lattice might affect the hydrodynamic behavior of the system. Figure 1 shows two lattice examples often used in two- and three-dimensional LB simulations. These lattices define 9 and 19 allowed velocities (including the quiescent state) and are thus named D2Q9 and D3Q19, respectively.

The densities $\Psi_{i}(r,t)$ are the elementary dynamical variables in LB. The macroscopic local density ρ(r,t) and velocity v(r,t) at position r can be evaluated based on $\Psi_{i}(r,t)$ as

(28) $$\rho \left(r,t\right)\;=\;\sum_{k}\Psi_{k}(r,t),$$

(29) $$\rho \left(r,t\right)\;v(r,t)\;=\;\sum_{k}{𝕧}_{k}\Psi_{k}(r,t),$$

in which the summation is performed over all allowed velocities. It is obvious that the local macroscopic properties can be evaluated with time, if the evolution of the particle distribution function is known. In LB the elementary two-step evolution (i.e., propagation and collision) of the particle distribution function after a time step ∆t can be written in a condensed format as

(30) $$\Psi_{i}(r\;+\;{𝕧}_{k}∆t\;,\;t\;+\;∆{t)\;=\;\Psi }_{i}(r,t)\;+\;\sum_{k}\Lambda_{ik}\left(\Psi_{k}{(r,t)\;-\;\Psi }_{k}^{\mathrm{eq}}(r,t)\right),$$

where the index k spans the velocity subspace, $\Psi_{k}^{\mathrm{eq}}(r,t)$ is the equilibrium distribution function and $\Lambda_{ik}$ is the collision matrix. The simplest form of the collision matrix was proposed by Bhatnagar, Gross, and Krook (BGK) as $\Lambda_{ik}\;=\;-\frac{1}{\tau }\delta_{ik}$ where τ is the collision time [216,217]. This method produces reasonably accurate solutions despite its simplicity [164]. The simplified form of Equation (30), i.e., the BGK-LB method, consequently is

(31) $$\Psi_{i}(r\;+\;{𝕧}_{k}∆t\;,\;t\;+\;∆{t)\;=\;\Psi }_{i}(r,t)\;+\;\frac{1}{\tau }\left(\Psi_{i}^{\mathrm{eq}}{(r,t)\;-\;\Psi }_{i}(r,t)\right).$$

The equilibrium distribution function $\Psi_{i}^{\mathrm{eq}}(r,t)$ needs to be defined before one can use Equation (31) to simulate a system. This is done by requiring that mass and momentum must be conserved [166]. A suitable form for the equilibrium distribution is often a quadratic function in velocities as [164]

(32) $$\Psi_{i}^{\mathrm{eq}}{\;=\;\rho w}_{i}\left[1\;+\;3\frac{{𝕧}_{i}\cdot v}{{𝕧}^{2}}\;-\;\frac{3}{2}\cdot \frac{v^{2}}{{𝕧}^{2}}\;+\;\frac{9}{2}\cdot \frac{{\left({𝕧}_{i}\cdot v\right)}^{2}}{{𝕧}^{4}}\right].$$

Here, $𝕧\;=\;\sqrt{3}\;{𝕧}_{s}$ where ${𝕧}_{s}$ is the speed of sound, and $w_{i}$ is the weighting constant. For D2Q9 lattice, $w_{i}$ is

(33) $$w_{i}\;=\{\begin{array}{cc} \frac{4}{9} & \mathrm{for}\;i\;=\;0\\ \frac{1}{9} & \mathrm{for}\;i\;=\;1,\;2,\;3,\;4\\ \frac{1}{36} & \mathrm{for}\;i\;=\;5,\;6,\;7,\;8 \end{array}\;\mathrm{and}\;\left|{𝕧}_{i}\right|\;=\{\begin{array}{cc} 0 & \mathrm{for}\;i\;=\;0\\ 𝕧 & \mathrm{for}\;i\;=\;1,\;2,\;3,\;4\\ \sqrt{2}𝕧 & \mathrm{for}\;i\;=\;5,\;6,\;7,\;8 \end{array},$$

and for D3Q19 lattice, it is defined as

(34) $$w_{i}\;=\{\begin{array}{cc} \frac{1}{3} & \mathrm{for}\;i\;=\;0\\ \frac{1}{18} & \mathrm{for}\;i\;=\;1,\;2,\;\ldots ,\;6\\ \frac{1}{36} & \mathrm{for}\;i\;=\;7,\;8,\;\ldots ,\;18 \end{array}\;\mathrm{and}\;\left|{𝕧}_{i}\right|\;=\{\begin{array}{cc} 0 & \mathrm{for}\;i\;=\;0\\ 𝕧 & \mathrm{for}\;i\;=\;1,\;2,\;\ldots ,\;6\\ \sqrt{2}𝕧 & \mathrm{for}\;i\;=\;7,\;8,\;\ldots ,\;18 \end{array}.$$

In the algorithm of BGK-LB method, one also needs to provide precise description of the boundaries of the system [164,165]. The discrete distribution function of LB on the boundaries has to be taken carefully so that it represents correct macroscopic boundaries of the system. LB has found various applications in polymer science [218], for instance, polymer solutions [133,178,219,220], simulation of complex flows [221,222], polymer electrolyte fuel cells [223], liquid crystals [224,225,226], deformation of droplets containing polymers and nanoparticles [227], and thermal conductivity and permeability of fibrous materials [228,229].

### 2.4. Macroscale Techniques

At the macroscopic scale, it is a common practice to disregard the discrete atomistic and molecular structures and assume that the material is continuously distributed throughout its volume. This approach is applicable provided that the behavior of the collections of atoms and molecules of the materials can be homogenized based on a proper understanding of the structures at the finer scales. Consequently, this scale is often referred to as the continuum scale in the literature. The continuum material is often assumed to possess average physical properties such as density, heat capacity, thermal conductivity, etc. and can be subjected to body forces such as gravity and surface forces such as contact between two bodies.

In general, the macroscale methods obey several fundamental laws [2,30]. These laws are (i) conservation of mass; (ii) equilibrium, based on Newton’s second law; (iii) the moment of momentum law, in which the moment is equal to the time derivative of angular momentum with respect to a reference point; (iv) conservation of energy; and finally (v) the conservation of entropy. Although these principles define the fundamentals for a macroscale model, they still need to be completed with suitable constitutive laws and the equations of state to provide all the information necessary in order to solve a macroscopic problem. It is noteworthy that the derivation of proper constitutive equations for polymeric systems has been an intriguing topic ever since the viscoelasticity concepts were introduced [230]. Various models are put forward with advantages as well as shortcomings often as a result of being limited to a certain class of either polymer systems or phenomena. Moreover, the implementation of usually complex viscoelastic constitutive equations results in extremely heavy calculations.

The continuum models often lead to a set of partial differential equations. In simple cases, it might be possible to find a closed-form analytical solution for the problem. However, it is often necessary to utilize appropriate numerical approaches to evaluate the solution due to the complexity of the involved phenomena. Finite difference method (FDM) is the simplest numerical method developed so far from a mathematical point of view. This simplicity comes with the price of losing flexibility for use with complicated geometries and phenomena compared with more elaborate numerical schemes such as finite element method (FEM) and finite volume method (FVM). It should be emphasized that all of these approaches are merely mathematical methods to estimate the solution of a set of partial differential equations and do not include a definite physical meaning in their bare core. Hence, they are not solely limited to the macroscale phenomena and the founding ideas behind them can also be applied to other scales. These numerical schemes ultimately transform the set of partial differential equations into a system of linear algebraic equations and solve it using either direct approaches, such as Gauss’ method, or iterative approaches, such as Gauss-Seidel method [231].

It should be noted that the macroscale techniques do not always deal with a continuous medium. For instance, smoothed particle hydrodynamics (SPH) is one such particle-based method which has been applied to study a number of phenomena including viscoelastic flows [232,233]. Moreover, the thermodynamically consistent version of SPH is named smoothed dissipative particle dynamics (SDPD) and has been implemented in multiscale frameworks to link the macroscopic SPH to the mesoscopic DPD method [234,235,236]. In its essence, SPH utilizes particles moving with the flow which make it possible to evaluate hydrodynamic properties at particle positions by a weighted averaging of the local values. Therefore, every particle is practically “smoothed” over a finite volume with fixed mass. For this part of the paper, we focus our attention to two widely-used mathematical methods in macroscale calculations, i.e., FEM and FVM.

#### 2.4.1. Finite Element Method

FEM is a powerful method to solve equations in integral form. Two possibilities exist for the application of FEM. In the first case, there exists an integral form of the physical problem. This integral form can be a result of a variational principle, the minimum of which corresponds to the solution, or more generally an integral equation to solve [231]. In the second case, an integral formulation must be obtained from an initial system of partial differential equations by a weak formulation, also called the weighted residual method [231].

A prerequisite of utilizing FEM is to decompose the spatial domain under consideration into a set of elements of arbitrary shape and size. This discretization is often called a grid or a mesh. In the decomposition procedure, the only restriction is that elements cannot overlap nor leave any zone of the domain uncovered. The definition of a mesh for FEM is more free compared with FDM for which the grid follows a coordinate system. For each element in FEM, a certain number of points, called nodes, must be defined which can be situated either on the edges of the element or inside it. The nodes are then used to construct the approximations of the functions under consideration over the entire domain by interpolation.

The approximation of a function u(r), where r is the vector of spatial coordinates, on a geometric domain meshed with finite elements is obtained as a linear combination of interpolation functions $\psi_{n}(r)$ associated with the mesh. If $u_{h}(r)$ is the approximation of the function u(r) under consideration, it can be expressed in the form of a sum over the nodes of the domain by

(35) $$u_{h}\left(r\right)\;=\;\sum_{n=1}^{N}u^{n}\psi_{n}(r),$$

in which N is the total number of nodes. The interpolation functions $\psi_{n}(r)$ can be of diverse forms with different degrees of continuity and differentiability. In the standard FEM, these functions are defined locally at the level of each element. Therefore, if the node n belongs to element e, and if $\psi_{n}^{e}$ is used to denote the restriction of $\psi_{n}$ within the element, for every coordinate vector r outside the element e, one has

(36) $$\psi_{n}^{e}\left(r\right)\;=\;0,$$

and for every coordinate vector r inside the element e,

(37) $$u_{h}\left(r\right)\;=\;\sum_{n\;=\;1}^{N}u^{n}\psi_{n}(r)=\;\sum_{n\in \;e}u^{n}\psi_{n}^{e}\left(r\right).$$

The last sum is performed only over the nodes that constitute the element e. Consequently, the interpolation used for approximation is locally defined at the level of each finite element. This way of decomposition and approximation thus distinguishes the standard FEM from other methods using interpolation functions defined over the entire domain. Moreover, in the standard FEM, the coefficients $u^{n}$ are the values of the function $u_{h}$ at the nodes of the mesh. As a result, the interpolation functions must satisfy two conditions in addition to Equation (36). First, if n and p are two nodes of the same element e, and $r^{p}$ is the position vector of the node p, then

(38) $$\psi_{n}^{e}\left(r^{p}\right){\;=\;\delta }_{np},$$

where $\delta_{np}$ is the Kronecker delta function. Second, to exactly represent constant functions, for all r inside the element e including the borders

(39) $$\sum_{n\in \;e}\psi_{n}^{e}\left(r\right)=\;1.$$

In most cases, the integral form of the problem should be also constructed from partial differential equations. For a simple case where the problem is limited to solve one partial differential equation of the form R(u) = 0 on domain Ω, one can utilize the weighted residual method to obtain the equivalent integral form. In the context of FEM, R(u) is often called the residual value. Obviously, the solution of the problem zeros the residual and simultaneously satisfies the boundary conditions at ∂Ω. The basic idea in FEM is to search for functions u which zero the integral form

(40) $$\Phi \left(u\right)=\int_{\Omega }\varrho \;R\left(u\right)\;\mathrm{dV}=\;0,$$

for every weighting function ϱ belonging to a set of functions $\left\{S_{\varrho }\right\}$, while u satisfies the boundary conditions at ∂Ω. The equivalence between R(u) = 0 on Ω and Equation (40) is only true if the set $\left\{S_{\varrho }\right\}$ has infinite dimensions and is composed of independent functions [231]. Otherwise, if $\left\{S_{\varrho }\right\}$ is finite as in FEM, the solution u which satisfies Equation (40) is only an approximate solution to the problem.

It should be noted that the weighted residual method is not the only method which can be used to search for a function that zeros the residual R(u) on Ω. For instance, the least-squares method can be applicable in some cases despite its limitations. The principle of least-squares consists of searching for the function u that minimizes the integral

(41) $$f\left(u\right)\;=\;\int_{\Omega }{\left(R(u)\right)}^{2}\;\mathrm{dV},$$

and that respects the boundary conditions. However, it is often difficult to employ the boundary conditions in this formalism. Furthermore, the order of derivatives in R cannot be reduced which leads to high differentiability conditions on the finite element discretization [231]. For these reasons, the method of weighted residuals is often preferred.

For the discretization of the obtained integral form, N independent weighting functions $\varrho_{1}$, $\varrho_{2}$, $\varrho_{3}$, …, $\varrho_{N}$ are utilized. There are different approaches to define the type of $\varrho_{i}$ functions. The most used approach is the Galerkin method which defines the weighting functions precisely the same as the interpolation functions $\psi_{n}$ of the approximation by finite elements [231]. Therefore, Equation (40) can be written as

(42) $$\Phi \left(u\right)=\int_{\Omega }\psi_{n}\;R\left(\sum_{n=1}^{N}u^{n}\psi_{n}\right)\;\mathrm{dV}=\;0.$$

This integral equation is later turned into a sum of finite series over the nodes of the domain. The boundary conditions are usually implemented into this integral form benefitting from the divergence theorem [231]. In the algorithm of FEM, for every element e a mapping can be defined between the element in physical space and a reference element, which allows defining the interpolation functions universally for the diverse elements regardless of their coordinates [231]. This notion facilitates programming profoundly.

FEM has been implemented in several simulation packages and consequently can be easily used by both academic and industrial communities, in a variety of applications. To name a few instances in polymer science, we note the prediction of the failure behavior of adhesives [237,238], the study of elastic modulus of polymer/clay nanocomposites [239], the prediction of temperature distribution in a tissue-mimicking hydrogel phantom during the application of therapeutic ultrasound [240], the wall slippage in the extrusion of highly-filled wood/polymer composites [241,242], the torsional friction behavior in hydrogels [243], permeation analysis in polymer membranes [244], viscoelastic flow analysis [245,246,247], and droplet deformation [248]. A significant improvement of the precision of FEM was achieved by Patera [249] when it was combined with spectral techniques. The resulting algorithm is generally known as the spectral element method (SEM). SEM is more stable and accurate than FEM under a relatively broad range of conditions [250]. Due to its power and versatility, SEM has shown to be a promising candidate to solve the viscoelastic models in the simulations of complex polymer flows [251,252].

#### 2.4.2. Finite Volume Method

FDM and FEM are admittedly the two most important classes of numerical methods for partial differential equations. However, they both suffer from serious shortcomings. The main defects of FDM are: (i) the considerable geometrical error of the approximation of curved domains by rectangular grids; (ii) the lack of an effective approach to deal with natural and internal boundary conditions; and (iii) the difficulty to construct difference schemes with high accuracy unless the difference equation is allowed to relate more nodal points and thus further complicating the incorporation of boundary conditions.

Classic FEM methods, i.e., Galerkin FEM (GFEM), perform successfully in fields such as solid mechanics and heat conduction where the problem is governed by self-adjoint elliptic or parabolic partial differential equations. Unfortunately, this success did not continue in the field of fluid dynamics. The reason was ascribed to the convection operators in the Eulerian formulation of the governing equations which render the system of equations non-self-adjoint [253]. Consequently, solutions to non-self-adjoint fluid dynamic problems by GFEM often suffer from node to node oscillations. This problem has motivated the development of alternatives to the GFEM which preclude oscillations without requiring mesh or time step refinement. The streamline-upwind/Petrov-Galerkin (SUPG) [254,255] and the least-squares finite element [231,256] methods are two examples of such approaches. Some authors also attempted to develop a strategy in FEM which employs a least-squares method for first-order derivatives and a Galerkin method for second-order derivatives in the governing Navier-Stokes equations [257]. Nevertheless, the simplicity of calculations and development of simulation algorithms is usually hindered by such approaches.

As a result, the search for a simple yet accurate alternative to FEM was carried out benefiting from FDM concepts and coupling it with finite element spaces in order to derive the so-called generalized differences methods (GDM) [253]. GDM provides several advantages such as small geometrical errors, easy handling of natural boundary conditions, and maintaining conservation of mass. With GDM, one is supplied with a method with the computational effort greater than classic FDM and less than FEM while the accuracy is higher than FDM and nearly the same as FEM. Due to its advantages, in particular its inheritance of the mass conservation law, GDM was rapidly developed in computational fluid dynamics (CFD) most popularly called FVM. FVM is also referred to as the finite control volume method which is a discrete estimation of a certain control equation in an integral form [258,259,260]. Hence, FVM is basically equivalent to GDM with piecewise constants and piecewise linear elements. Using FVM to develop numerical algorithms for nonlinear equations is in fact generalizing the classical difference schemes to irregular meshes. The equivalence of FDM and FVM has been shown in simple cases for instance by Rappaz et al. [231].

Although FVM has been applied to many applications including magnetohydrodynamics [261,262,263], structural dynamics [264,265], and semiconductor theory [266,267], its main field of application has been CFD mainly due to its conservative nature. Consequently, we restrict ourselves to this field in the rest of this section. Similar to FDM and FEM, FVM changes a set of partial differential equations with a system of linear algebraic equations. In order to do this, FVM utilizes a two-step discretization procedure [268]. First, the partial differential equations are transformed into balance equations by integration. In this transformation the surface and volume integrals are changed into discrete algebraic equations over individual elements benefitting from an integration quadrature. A set of semi-discretized equations is then produced. Second, the local values of the variables in the elements are approximated by using suitable interpolation profiles. For a general scalar variable ϑ, one can write the steady state conservation equation as

(43) $$\nabla \cdot \left(\rho v\vartheta \right)\;=\;\nabla \cdot \left(D^{\vartheta }\nabla \vartheta \right){\;+\;Q}^{\vartheta },$$

where ρ is the fluid density, v is the fluid velocity vector, $D^{\vartheta }$ is the diffusion coefficient of ϑ, and $Q^{\vartheta }$ is the generation/destruction of ϑ in the control volume per unit volume. By integrating the above equation over the element e and utilizing the divergence theorem, one finds

(44) $$\oint_{\partial V_{e}}\left(\rho v\vartheta \right)\cdot dS\;=\;\oint_{\partial V_{e}}\left(D^{\vartheta }\nabla \vartheta \right)\cdot dS\;+\;\int_{V_{e}}Q^{\vartheta }\;\mathrm{dV},$$

in which S represents the surface vector, and $\partial V_{e}$ shows that the integration is performed over all the surfaces surrounding the volume $V_{e}$. The semi-discrete steady state equation for e can be finally simplified to [268]

(45) $$\sum_{\varepsilon \sim \mathrm{neighboring}\;\mathrm{cells}\;\mathrm{of}\;e}\;{\left(\rho v\vartheta {\;-\;D}^{\vartheta }\nabla \vartheta \right)}_{\varepsilon }\cdot S_{\varepsilon }{\;=\;Q}_{\varepsilon }^{\vartheta }V_{\varepsilon },$$

by using the mid-point integration approximation. The summation is performed over the faces surrounding element e with its neighboring cells. Here, $Q_{e}^{\vartheta }$ is the contribution of element e to $Q^{\vartheta }$. If one denotes the convection and diffusion flux terms by $J^{\vartheta ,C}$ and $J^{\vartheta ,D}$, respectively, one can write Equation (45) in the form

(46) $$\sum_{\varepsilon \sim \mathrm{neighboring}\;\mathrm{cells}\;\mathrm{of}\;e}\;{(J^{\vartheta ,C}+J^{\vartheta ,D})}_{\varepsilon }\cdot S_{\varepsilon }{\;=\;Q}_{\varepsilon }^{\vartheta }V_{\varepsilon },$$

where $J^{\vartheta ,C}\;=\;\rho v\vartheta$ and $J^{\vartheta ,D}{\;=\;-D}^{\vartheta }\nabla \vartheta$. In FVM, the transported variable ϑ is conserved in the discretized solution domain since the fluxes at a face of an element are calculated using the values of the elements which share that face [268]. As a result, for any mutual surface of two elements, the outwards flux from a face of an element is precisely equal to the inwards flux from the other element through that same face. Consequently, such fluxes are equal in magnitude but with opposite signs.

To get the fully-discretized steady state finite volume equation for element e, one needs to adjust proper interpolation profiles. The interpolation profiles are often different for diffusive and convective terms due to the distinct physical phenomena that these terms represent. For the diffusive term, a linear interpolation profile is often used [268]. The selection of an interpolation profile for the convective terms could be more challenging. The simplest interpolation scheme, i.e., the symmetrical linear profile or the central difference scheme, could be applied here. Despite its simplicity, this scheme can result in unbounded unphysical behavior at high Peclet numbers (Pe) due to the fact that it cannot describe the directional preference of convection [268]. Consequently, the upwind scheme was introduced to account for this directional preference and provide a better stability at the cost of the accuracy. This is due to the fact that the upwind scheme has a first order of accuracy whereas the linear scheme has a second order of accuracy [269]. In order to enhance the precision and stability of advection schemes, higher-order upwind biased interpolation profiles were incorporated in the calculations. Such higher-order schemes often produce at least a second-order accurate solution, while they are unconditionally stable. An example of such attempts is the quadratic upstream interpolation for convective kinematics (QUICK) scheme developed by Leonard [270]. In this method, the value of the dependent variable is interpolated at each element face using a quadratic polynomial biased towards the upstream direction. Further details can be found elsewhere [268].

In recent years, the application of FVM in CFD has been significantly accelerated, mostly because of the emerging open source software packages such as OpenFOAM^®^ (Open Source Field Operation and Manipulation) [271,272]. Analysis of viscoelastic fluids [273,274,275,276,277,278,279], viscoelastic two-phase flows [280], mold filling in water-assisted injection molding of viscoelastic polymers [281], gas permeation in glassy polymer membranes [282], blood flow [283], development of droplet and co-continuous binary polymer microstructures [284] are some examples of FVM applications in polymer science.

## 3. Multiscale Strategies

The ultimate purpose of a multiscale modelling is to predict the macroscopic behavior from the first principles at the quantum scale. Finding appropriate protocols for multiscale simulations is on the other hand a very challenging topic. This is due to the fact that polymeric materials often display phenomena on one scale that necessitate a precise description of other phenomena on another scale. Since none of the methods discussed before is sufficient alone to describe a multiscale system nor they are designed for such a purpose, the goal becomes to develop a proper combination of various methods specialized at different scales in a multiscale scheme. This scheme is also supposed to effectively distribute the computational power where it is needed most. By definition, such a multiscale approach can take advantage of the various methods it envelops at multiple scales and reaches the length and time scale that the individual methods fail to achieve. At the same time, this approach can retain the precision provided by the individual methods in their respective scales. Moreover, the multiscale approach should be flexible enough to allow for high accuracy in particular regions of the systems as required. Therefore, the overall objective of multiscale models is to predict the behavior of materials across all significant length and time scales while preserving a balance among precision, efficiency, and realistic description.

In general, there are three main categories of multiscale approaches: sequential, concurrent, and adaptive resolution schemes. The sequential approach links a series of computational schemes in which the operative methods at a larger scale utilize the coarse-grained (CG) representations based on detailed information attained from smaller scale methods. Sequential approaches are also known as implicit, serial, or message-passing methods. The second group of multiscale approaches, the concurrent methods, are designed to bridge the suitable schemes of each individual scale in a combined model. Such a model accounts for the different scales involved in a physical problem concurrently and incorporates some sort of a handshaking procedure to communicate between the scales. Concurrent methods are also called parallel or explicit approaches. It is noteworthy that multiscale simulations could principally utilize a hybrid scheme based on elements from both sequential and concurrent approaches. More recently, a new concept for multiscale simulations has been developed which resembles some characteristics of concurrent methods. In this approach, single atoms or molecules can freely move in the simulation domain and switch smoothly from one resolution to another, for instance based on their spatial coordinates, within the same simulation run. Consequently, these methods are generally referred to as the adaptive resolution simulations. Details of such techniques are provided in the following sections. Finally, there are a number of advanced techniques which allow for extending the reach of a single-scale technique such as MD within certain conditions. Such methods are also reviewed for the sake of completeness before closing the discussion of multiscale strategies.

### 3.1. Sequential Multiscale Approaches

In sequential approaches, calculations are often performed at a smaller scale (the more detailed, finer scale) and the resulting data are passed to a coarser model at a larger scale after leaving out unnecessary details for instance by coarse-graining. However, it will be shown that in some cases the reverse procedure can also be done. A sequential multiscale model requires a thorough understanding of the fundamental processes dominating the finest scale to yield accurate information. Afterwards, it is also crucial to have a well-founded approach to introduce this information into the coarser scales. Such a strategy is usually achieved by utilizing phenomenological theories which contain some key parameters. These parameters are then used as the linking bridges between the scales when their values are determined from the calculated data of the finer scale simulations. This message-passing method can be performed in sequence for multiple length scales. It is obvious that in this sequential approach the accuracy of the simulations at the coarser scale critically depends on the accuracy of the information from the finer scale simulations. Furthermore, the model at the coarser scale must be accurate itself so that it can provide reliable results. In this strategy, the relations between the scales must be invertible so that the results of the coarser scale simulations can be used to suggest the best choice for the finer scale parameters.

The sequential approach has generally proven effective in systems where the different scales are weakly coupled. Therefore, appropriate systems for such a methodology often share a common character by which the large-scale variations appear homogeneous and quasi-static from the small-scale perspective. The majority of the multiscale simulations that have been actually incorporated in materials research are in fact sequential. In order to highlight the sequential message-passing in a range of polymeric systems, a few examples are outlined here. To predict the morphology and mechanical properties of mixtures of diblock copolymers and rod-like nanoparticles, Shou et al. [285] coupled the self-consistent field theory with DFT to provide input information for the lattice spring model (LSM). In their sequential algorithm, the spatial morphology of different phases is mapped onto the coarser-scale lattice and the force constants are derived for the three-dimensional network of springs. In similar approaches, other methods including LB [286], MC [287], and MD [288,289], have also been used to produce appropriate morphological information for LSM in various systems including polymer blends and nanocomposite coatings. Recently, the classical fluids density functional theory was linked to MD simulations by Brown et al. [290] to study microphase separated states of both typical diblock and tapered diblock copolymers. The fluids density functional theory can predict the equilibrium density profiles of polymeric systems. The authors used the resulting density profiles of this theory to initialize MD simulations with a close to equilibrated structure and could speed up the simulations. In a study on the influence of self-assembly on the mechanical and electrical properties of PNCs, Buxton and Balazs [291,292] used a combination of Cahn-Hillard theory and BD at the finer scale to produce morphological data. The data were later fed either into LSM in order to determine the mechanical properties, or into FDM to calculate the electrical conductivity.

**Figure 2:**
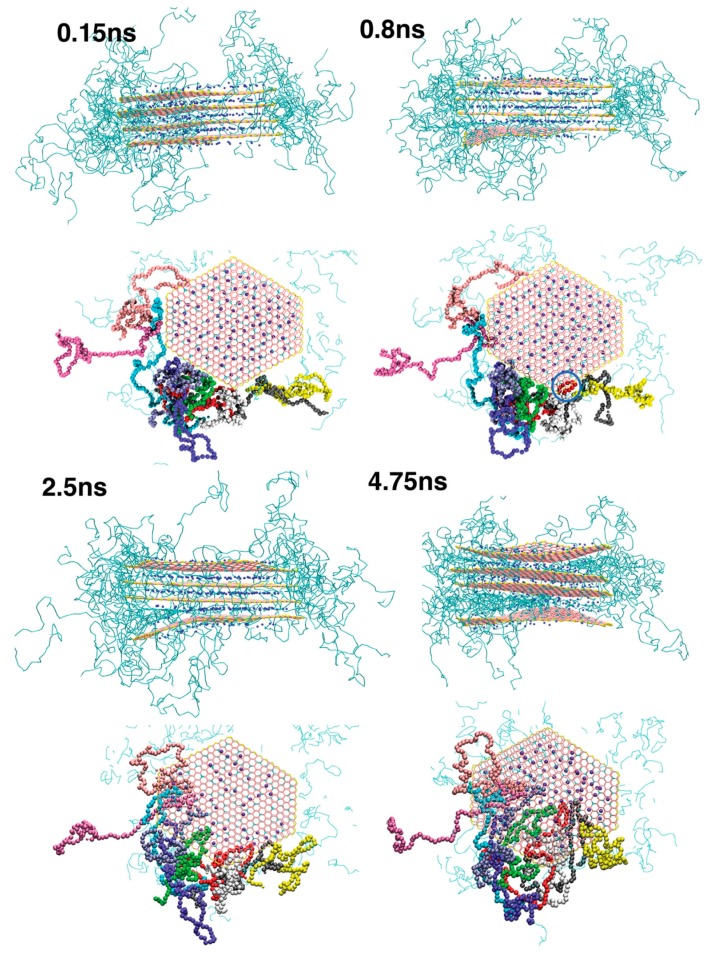
Pictorial overview of the intercalation of poly(vinyl alcohol) chains in a clay tactoid. The side and top views of the tactoids are illustrated at several snapshots. The macromolecules are shown by the green bonds in the side views. The color code for the clay particles are: pink: neutral clay; cyan: charged clay; yellow: edge of the clay; and blue: sodium. The bending of the lowermost clay due to the intercalation process of poly(vinyl alcohol) chains can be observed in the side view snapshots. For the top view, the intercalating polymers are colored based on their molecule number, to make the visualization easier. One can see that the polymer initially starts intercalating as short loops (for an instance see the blue circled chain at the 0.8 ns snapshot), and progresses further into the interlayer. Reprinted from Suter et al. [293] under the terms of the Creative Commons Attribution License.

**Figure 3:**
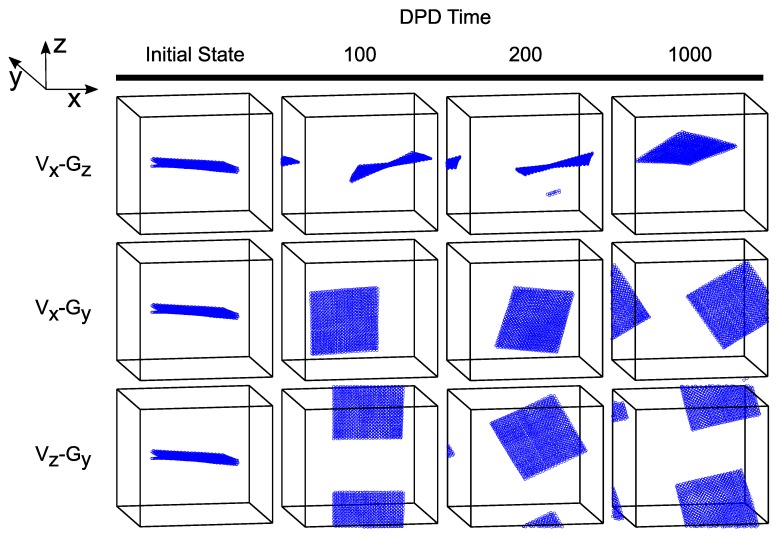
Snapshots of the clay platelets with time experiencing various flow directions. The applied shear-rate is 0.148 in DPD units and the flow of each row is defined in the figure; the velocity direction is shown by V_direction_ and the velocity gradient direction by G_direction_. Reprinted from Gooneie et al. [195]. Copyright 2016, with permission from John Wiley & Sons Inc.

A number of studies have been devoted to characterize polymer/clay nanocomposites at different scales, spanning from quantum mechanical scale up to the macroscale. One such algorithm was developed by Suter et al. [293] which starts with the quantum theory, and transfers the key information through atomistic classical MD to a CG representation. This sequential procedure allowed for the study of the intercalation of molten polymers, poly(ethylene glycol) and poly(vinyl alcohol), within MMT tactoids and the larger scale ordering of these bridged tactoids, see Figure 2. In a separate multiscale study, Scocchi et al. [294] evaluated the rescaled energies of a CG DPD model from the energy values of their atomistic MD counterparts. Using this information, they could calculate the maximum repulsion coefficients for the corresponding DPD models of polyamide (PA)/clay and polypropylene (PP)/clay nanocomposites and reproduce experimentally observed microstructures. The same methodology was also applied in following works and was extended into the macroscale realm by linking to FEM in order to derive mechanical properties of polymer/clay nanocomposites as a function of the degree of exfoliation [295,296]. The DPD parameters of their work derived from MD simulations, were recently shown to be capable to capture the orientation dynamics of clays in polymer melts under various shearing flows, see Figure 3 [195].

The most common serial transfer of information from a finer scale method to a coarser one can be envisioned in the systematic development of CG models of polymer systems. The CG models are often designed to reproduce the configurations of more detailed descriptions in atomistic simulations as accurately as possible. In this way, a CG model with much less degrees of freedom is achieved which can access longer time scales appropriate for instance in dynamics simulations. It is worthy to note that the final conformations of such CG simulations could be translated back to its atomistic details based on a specific backmapping algorithm. These sequential procedures represent general characteristics of sequential multiscale approaches and could also be extended to more complex systems. Furthermore, these fields have witnessed a large amount of research activities in recent years. As a result, more details are provided on these topics to help the reader familiarize oneself with the underlying challenges and possibilities.

#### 3.1.1. Systematic Coarse-Graining Methods

A serious problem with polymeric materials in a sequential multiscale scheme is that the coarse-graining method from atomistic scale to mesoscale or from mesoscale to macroscale is not a straightforward procedure. The coarsening from QM to MD follows basic principles which can be formulated in a computational framework while it is system-specific at higher scales. All methods are based on the application of a force field which transforms information from quantum scale to atomistic simulations. From atomistic simulations to mesoscale model, critical features of the system such as the structure and/or thermodynamics have to be preserved while the degrees of freedom is reduced. The linking of scales through the mesoscale is addressed by many authors as the most challenging step towards developing reliable multiscale frameworks. Systematic coarse-graining methods are therefore developed to address these challenges. It is noteworthy that some mathematical aspects of various coarse-graining methods for equilibrium [297] and nonequilibrium [298] systems were addressed recently in details.

Systematic coarse-graining strategies attempt to extend the length and time scales of atomistic MD simulations by replacing several atoms with a single super atom and thus reducing the degrees of freedom. These approaches strictly attempt to preserve intrinsic properties of polymers such as radius of gyration, diffusion coefficient, etc. As a consequence, the results of such CG models can be directly compared with experiments. Depending on the number of atoms that are lumped into a single super atom, i.e., the degree of coarse-graining, the systemic coarse-graining methods are roughly divided into three major blocks; (i) low coarse-graining degrees where one or two monomers are coarse-grained into one super atom; for instance, in an iterative Boltzmann inversion (IBI) scheme; (ii) medium coarse-graining degrees where ten to twenty monomers are coarse-grained into one blob or bead, for instance, used in the so-called “blob model”; and (iii) high coarse-graining degrees where the whole chain is mapped to a single soft colloid in super coarse-graining methods. These variations provide access to a range of time and length scales from 10^−6^ s (10^−6^ m) to 10^−2^ s (10^−2^ m), particularly precious to simulate dynamic properties of polymeric systems [299]. In addition to the reduced number of degrees of freedom, CG models often benefit from simpler forms of interactions compared with the detailed models. This feature can promote the computational efficiency to a large extend. Besides, the free energy profiles of CG models are usually smoother due to the fact that many interaction centers are replaced with only a single site. Finally, the parametrization of the CG interactions is simpler than that of full atomistic systems since many chemistry-specific details are ignored during coarse-graining. Such features of CG models make them particularly appealing for many applications in polymer systems. In the next sections, several methods for coarse-graining as well as various remaining challenges are discussed.

##### Low Coarse-Graining Degrees

Low degrees of coarse-graining with one or two monomers lumped into a single super atom are carried out by either parameterized or derived approaches [300]. The parameterized approaches utilize all-atomistic (AA) simulations to calculate some target property, such as a pair distribution function, and then the coarse-graining potentials are evaluated to reproduce the target quantities. One should note that the CG potentials can hardly reproduce all the original AA system specifications. On the other hand, in the derived methods the CG pair potentials are calculated in AA simulations from the direct interactions between the groups of atoms enveloped in super atoms. In these methods, the contribution of multibody interactions to the effective CG potentials is less significant in comparison with pair potentials. Consequently, the derived methods are often used to describe systems in which multibody interactions do not play a significant role. Examples of derived methods are the pair potential of mean force (pPMF) [301,302], the effective force CG (EFCG) [303], and the conditional reversible work (CRW) [300,304,305]. In the rest of this part, we focus on parametrized approaches since the derived methods are generally considered to be better-suited for small molecules even though they have recently found some applications in larger molecules [306,307].

The parameterized methods are divided into structure-based and force-based methods depending on the target quantities. As specified in the name, structure-based methods construct the CG potentials in order to reproduce a structural property of the AA system such as pair distribution functions [36,308,309,310,311,312,313,314,315,316,317,318]. The IBI method is undoubtedly the most significant example of such methods [308,319]. Other structure-based methods include the Kirkwood-Buff IBI method [320], the inverse Monte Carlo (IMC) method [309,310,313], the relative entropy method [321,322,323,324], and the generalized Yvon-Born-Green theory [325]. All of these methods are principally similar to the IBI method with minor differences in their optimization or mapping schemes. The force-based approaches, on the other hand, attempt to match the force distributions on a super atom from both the CG and AA representations. There are mainly two variations to force-based methods namely the force-matching method [3,326,327,328,329,330,331], and the multiscale coarse-graining method [328,329,332,333,334,335]. For the sake of completeness, we should mention that in some works a combination of the methods is used to derive the CG model. For instance, we refer to the recent study of Wu [336] who utilized a combination of IBI and CRW to find the CG potentials for morphological simulations of poly(vinyl chloride)/poly(methyl methacrylate) and PS/poly(methyl methacrylate) blends.

In the IBI method, one often assumes that the probability distribution function $p^{R}$ depends on pair distance r, bond length l, bond angle θ, and dihedral angle ℧. These parameters are further taken to be independent from each other so that $p^{R}\left(r,l,\theta ,℧\right){\;=\;p}^{R}\left(r\right){\;\times \;p}^{R}\left(l\right){\;\times \;p}^{R}\left(\theta \right){\;\times \;p}^{R}\left(℧\right)$ and the CG potential function becomes $U^{\mathrm{CG}}\left(r,l,\theta ,℧\right){\;=\;U}^{\mathrm{CG}}\left(r\right){\;+\;U}^{\mathrm{CG}}\left(l\right){\;+\;U}^{\mathrm{CG}}\left(\theta \right){\;+\;U}^{\mathrm{CG}}\left(℧\right)$. Through the simple Boltzmann inversion one has $U^{\mathrm{CG}}\left(q\right){\;=\;-k}_{B}{T\;\ln\;p}^{R}\left(q\right)$ with q = r,l,θ,℧. The iterative algorithm in IBI compares the probability distribution functions of the CG model with the corresponding target probability distribution functions of AA simulations $p_{\mathrm{target}}^{R}$, and improves the calculated CG potential functions in a step-wise manner according to [299,337,338].

(47) $$U_{i+1}^{\mathrm{CG}}{(q)\;=\;U}_{i}^{\mathrm{CG}}{(q)\;+\;k}_{B}T\;\ln\frac{p_{i}^{R}(q)}{p_{\mathrm{target}}^{R}(q)}$$

The potential correction term, i.e., the second term on the right hand side of the equation, is sometimes multiplied by a relaxation factor between zero and one to avoid overshooting in the numerical procedure. The number of iterations required to reach satisfactory property reproduction in IBI is system-specific and depends on various factors like polymer structure, the definition of the super atom, the degree of coarse-graining, etc. and can take from a few to hundreds of iterations to converge [327]. Li et al. [339] used such a strategy to reproduce viscoelastic properties of *cis*-polyisoprene. In their work, the authors reproduced CG distribution functions and those obtained from AA simulations. In this way, they could optimize the potential functions for the four independent parameters separately.

The IBI method is not the only way to optimize a CG model based on AA simulations. Here we take a quick look at two other methods namely IMC and force-matching methods. IMC or the Newton inversion method incorporates rigorous statistical mechanical arguments to update the potential functions of the CG model [309,310,313]. The optimization procedure in IMC poses an interdependent updating algorithm for pair potentials in multicomponent systems whereas in IBI method these potentials are updated separately which could lead to convergence problems. However, this feature is often computationally very expensive [327]. In the force-matching method, a variational approach is used to construct the CG potentials based on the recorded forces from AA simulations [3,326,327,328,329,330,331]. In this method, the difference between the average AA force on a particle and the corresponding force in the CG counterpart is minimized in order to find the optimized CG force field. Thus, the force-matching approach actually projects the full many-body force field onto the definitive potential functions of the CG force field [340]. Due to the fact that the CG force field is merely an approximation of the AA force field, the force-matching method may or may not reproduce the structural properties of the AA system perfectly. The incorporation of higher-order interactions in the definition of the CG force field could resolve this problem at the cost of lower computational efficiencies [341]. It should be noted that IBI and similar methods are usually not helpful in systems with a diluted component since the interactions between the diluted molecules cannot be readily obtained. In such cases one should compute the effective potentials for these interactions with more rigorous sampling schemes such as thermodynamic integration or umbrella sampling [306,342,343,344].

**Figure 4:**
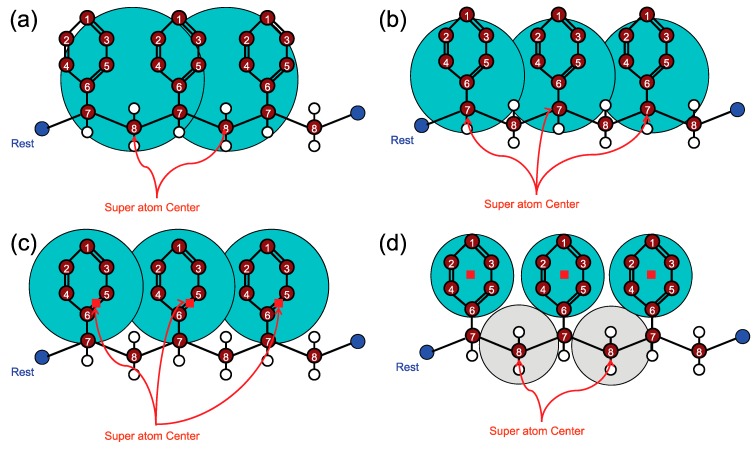
Different definitions for the super atoms of CG PS utilized by (**a**) Müller-Plathe and co-workers [347,348,349]; (**b**) Sun and Faller [351,352]; (**c**) Qian et al. [353]; and (**d**) Harmandaris et al. [354,355]. Reprinted from Li et al. [299] under the terms of the Creative Commons Attribution License.

In the coarse-graining procedure, there is usually more than one way to define super atoms. Several important issues regarding the definition of super atoms should be addressed carefully, i.e., the shape of the super atom, the position of the center of a super atom on a molecule, the number of atoms which are enveloped by it, as well as the number of different super atoms associated with a molecule. The super atom is defined to be a spherical particle in most studies, but there are also some works which offer generalizations for anisotropic potentials [345,346]. This enforces additional complexity on the definition of potential functions as well as the performance of CG simulations only for a slightly increased accuracy. Therefore, it is generally advised to achieve higher precisions by incorporating additional spherical super atoms to characterize the molecules instead of utilizing non-spherical super atoms [299]. Considering the other parameters mentioned for the definition of super atoms, there is no general rule applicable for different cases. There are various ways to define the super atoms to represent a CG model of a system. However, it is crucial to ensure that the final CG model is capable to reproduce the static, dynamic or thermodynamic properties correctly before it is further applied. To give an example, we consider the various possibilities to develop CG models of polystyrene (PS), which has been extensively studied with different approaches in the definition of super atoms as illustrated in Figure 4. Müller-Plathe and his co-workers [347,348,349] adopted the CG structure shown in Figure 4a and could successfully reproduces the gyration radius and the Flory characteristic ratio of PS in melts at 500 K. Nevertheless, the entanglement length was estimated to be much smaller than the experiments. Spyriouni et al. [350] modified the CG potential functions of this model and could predict the correct entanglement length of PS melts as well as the packing length and the tube diameter. Still, the isothermal compressibility was largely different from experimental values indicating the poor transferability of the developed potentials to pressures other than the one used in AA simulations. Another CG representation was developed by Sun and Faller [351,352] as depicted in Figure 4b which could obtain the entanglement length at 450 K in agreement with experimental observations. The mapping scheme shown in Figure 4c was developed by Qian et al. [353] which yields potentials capable of reproducing the isothermal compressibility as well as structural properties of the PS melts from 400 to 500 K. Finally, in order to include the tacticity effects on the structural and dynamic properties of PS, Harmandaris et al. [354,355] and Fritz et al. [356] used the CG models shown in Figure 4d. This model has been applied to study both the mechanical properties of PS glasses [357,358] and the dynamic properties of PS melts [359,360]. These works manifest the influence of the definition of super atoms on the final outcome of the simulations. Consequently, a CG model should be tested and validated for its predictive features and merits before any further use [361].

The fact that several atoms are replaced with a super atom in CG models changes the entropy due to the deleted degrees of freedom. This leads to an altered internal dynamics after coarse-graining. This notion becomes more important as the degree of coarse-graining increases. In addition to this altered entropy, the coarse-graining procedure changes the amount of the surface of each molecule available to its surrounding molecules due to the fact that it simplifies a cluster of atoms into a spherical super atom. Consequently, the hydrodynamic radius of the CG super atom is strongly dependent on the coarse-graining methodology and in every case, it is different from its AA counterparts. Since the friction coefficient is related to the hydrodynamic radius according to Stokes’s law [362], the coarse-graining procedure also changes the internal friction coefficient between monomers which leads to incorrect dynamic behavior of CG models [363,364,365]. Therefore, it is necessary to rescale the dynamics in order to simulate the correct behavior [366]. The dynamic rescaling can be performed utilizing a time-mapping factor defined, for instance, as the ratio of the friction coefficients [359,360], the ratio of decorrelation times utilizing the autocorrelation function [339], or numerically derived from the ratio of the mean square displacements (MSD) [354], between AA and CG models. In spite of these efforts, the correct definition of a time-mapping factor is still a challenge due to the fact that different modes of motions in a system should be scaled with different characteristic scaling factors, giving rise to the so-called “dynamical heterogeneity” issue [367,368,369].

Finally, the transferability and thermodynamic consistency of developed CG models should be ensured. In a coarse-graining procedure such as IBI, the effective potential functions are often evaluated based on target distribution functions, which are themselves derived for a specific set of thermodynamic conditions resembling a certain ensemble. Therefore, the derived potential functions from one state are not transferable to another state in most cases [337,370]. All CG models are state-dependent and should not be transferred to another state without re-parametrization. The “state” contains information about temperature, density, concentration, system composition, phase, etc. as well as chemistry-specific details of the system. An example for the thermodynamic inconsistency of CG models and AA simulations is the missing long-range interactions between the super atoms leading to overestimations of the pressure. To compensate for such effects, some studies add a linear attractive tail function into the pair potential and recover the correct pressure for CG polymer systems [319,371,372]. Consequently, the effective potential functions should be optimized individually for each state of the CG system. Despite this general consideration, there are some instances in the literature where the effective potential functions of the CG model possess a range of transferability into a subset of thermodynamics states [353,373,374,375]. For instance, the effective CG potentials of homopolymer melts show a remarkable transferability over a large range of temperatures [376,377,378]. Such studies state that the definition of super atoms largely influences the transferability of the effective CG potentials derived by the IBI method. An interesting topic in the transferability of CG models is to find a methodology to derive CG potentials which are both thermodynamically and structurally consistent with the underlying AA description [317,318,338,344,379,380,381,382]. Such a method could ensure a certain state transferability for the constructed CG potentials. Using calibration methods in order to improve the transferability of derived CG potentials is also an interesting possibility. Recently, inspired by ideas from uncertainty quantification and numerical analysis, Patrone et al. [383] used a Bayesian correction algorithm [384] to efficiently generate transferable CG forces. Their method uses functional derivatives of CG simulations to rapidly recalibrate initial estimates of forces anchored by standard methods such as force-matching.

##### Medium Coarse-Graining Degrees

**Figure 5:**
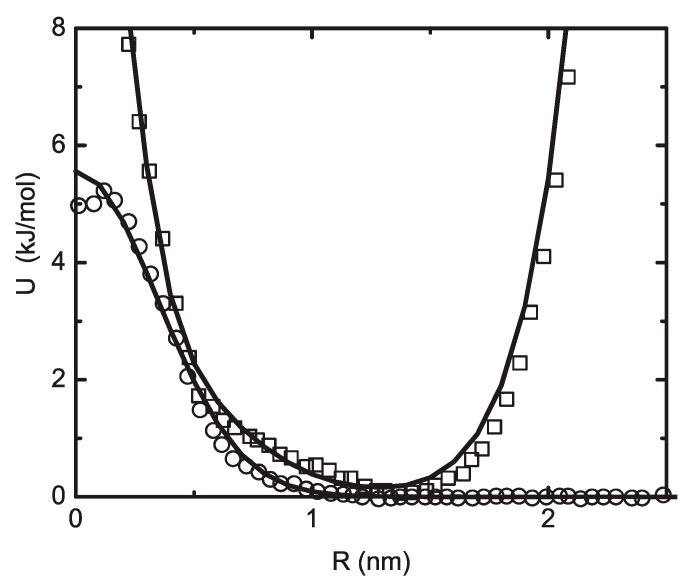
Potential functions for nonbonded (circles) and bonded (squares) interactions from AA simulations. The solid lines are fitted with Equations (48) and (49). Reproduced from Padding and Briels [385] with the permission of AIP Publishing.

**Figure 6:**
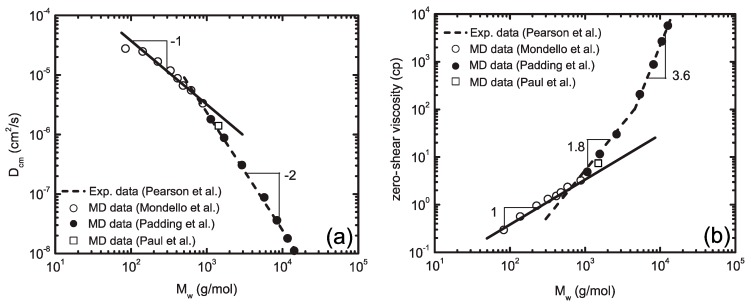
(**a**) Center-of-mass self-diffusion coefficient, $D_{\mathrm{cm}}$; and (**b**) zero-shear viscosity versus molecular weight, $M_{w}$ , for PE melts at 450 K. Reproduced from Padding and Briels [386] with the permission of AIP Publishing. For further information regarding the various sets of data shown in figure refer to the cited work and the references within it.

Since the definition of the super atom is not unique, it is possible to lump several monomers of the polymer chain into one single super atom. In this way, the approachable length and time scales of the CG simulations are significantly extended. Based on this idea, Padding and Briels lumped 20 monomers along a PE chain in a single spherical blob and developed the so-called “blob model” [385,386,387]. The potential functions of the blob model are optimized systematically based on AA simulations in a similar fashion to IBI. However, due to the larger number of lumped monomers in comparison with techniques for low coarse-graining degrees, the dihedral interactions between the blobs are negligible. Therefore, the potential functions of the blob model usually consist of nonbonded and bonded (i.e., bonds and bond angles) interactions. Padding and Briels write these interactions as

(48) $$U_{\mathrm{nonbonded}\;}^{\mathrm{CG}}{(r)\;=\;c}_{0}e^{-(\frac{r}{b_{0}}{)}^{2}},$$

(49) $$U_{\mathrm{bond}\;}^{\mathrm{CG}}{(l)\;=\;c}_{1}e^{-(\frac{r}{b_{1}}{)}^{2}}{\;+\;c}_{2}e^{-(\frac{r}{b_{2}}{)}^{2}}{\;+\;c}_{3}l^{\mu },$$

(50) $$U_{\mathrm{angle}\;}^{\mathrm{CG}}{(\theta )\;=\;c}_{4}{\left(1\;-\cos\theta \right)}^{\nu },$$

in which $U_{\mathrm{nonbonded}\;}^{\mathrm{CG}}(r)$, $U_{\mathrm{bond}\;}^{\mathrm{CG}}(l)$, and $U_{\mathrm{angle}\;}^{\mathrm{CG}}(\theta )$ are the potentials of nonbonded, bond and angle interactions, respectively. $c_{0}$ to $c_{4}$, $b_{0}$ to $b_{2}$, μ and ν are fitting parameters derived from AA simulations. The potential functions for nonbonded and bonded interactions Equations (48) and (49), respectively are optimized against AA results for the blob representation of PE illustrated in Figure 5. Blob model has been applied in a number of studies including the investigation of transient and steady shear flow rheological properties of polymer melts [388], chain dynamics of poly(ethylene-*alt*-propylene) melts [389], and entangled star PE melts [390]. In the blob model, it is also necessary to rescale the dynamics to capture the behavior of the polymer chains correctly. The rescaling can be performed by adjusting the friction coefficient of the Langevin equation to the simulated value from the AA model [386]. Based on this rescaling strategy, the correct diffusion coefficients and scaling laws of the zero-shear viscosity of PE polymer melts were predicted correctly in the blob model as shown in Figure 6 [386].

**Figure 7:**
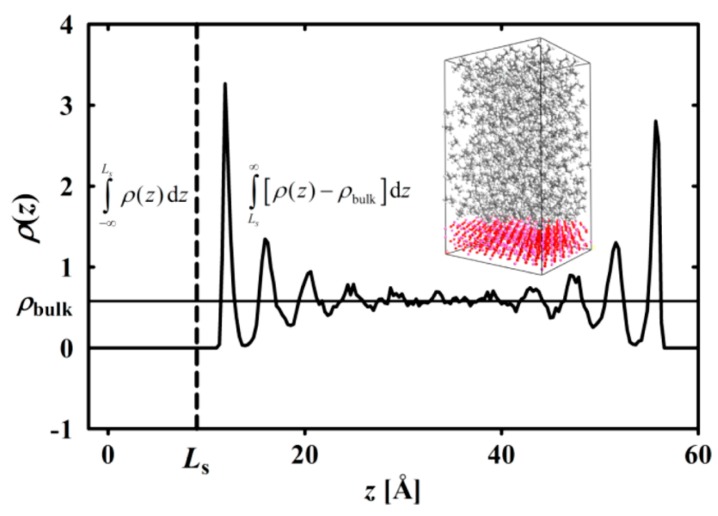
Number density profile from atomistic MD simulations. Molecular center-of-mass of a particular bead is used in computation of the profiles. Vertical line is the location of the substrate surface and defines the integration boundaries. A pictorial representation of the atomistic simulation box snapshot is given as the inset picture. Reprinted with permission from Kacar et al. [203]. Copyright 2016 American Chemical Society.

Another exciting method used to perform CG simulations with medium coarse-graining degrees is DPD which was introduced in Section 2.3.2. The conservative force in DPD algorithm was shown by Groot and Warren [187] to be connected to the Flory-Huggins parameters between components. This notion was further generalized to consider bead-size effects [391], variable bead volumes [392], as well as polymer blends [200]. The consideration of variable bead volumes in DPD facilitates the way to simulate more complex polymeric systems where beads can represent various functional chemical units with different volumes rather than polymers constructed from a single bead type [202]. In addition, an elaborate systematic strategy for parameterization of chain molecules in DPD simulations was recently proposed by Lee et al. [205] which successfully combines top-down and bottom-up approaches and benefits from experimental infinite dilution solubilities of the compounds to map the repulsion interaction parameters. There are rather simple relationships in the literature using which one can find the appropriate DPD conservative forces for all-fluid systems [202,203]. However, such relations cannot help in DPD studies where a fluid is interacting with a solid substrate. As a consequence, some authors developed an iterative approach to optimize the repulsive forces of DPD versus AA simulations based on a comparison of the density profiles of fluid particles on the solid substrate [201,202,203]. An example of such analysis is shown in Figure 7 for the parametrization of epoxy-alumina interactions as utilized by Kacar et al. [203]. A similar coarse-graining strategy was also incorporated by Johnston and Harmandaris [393] to study model polystyrenes on a gold surface. In their methodology, the authors developed a hierarchical multiscale model in which DFT, MD, and CG models were combined to describe the interfacial properties.

The distribution functions become broader as more atoms are coarse-grained into one super atom since more degrees of freedom are smeared out through averaging. Accordingly, the potential interactions become increasingly soft and therefore unphysical bond-crossings may occur in such systems. Such bond-crossings result in unrealistic predictions of the dynamics in the modelling of long polymer chains by reducing the number of entanglements. Hence, it is important to avoid the bond-crossing phenomenon in CG models. There are three main routes available to avoid (or to reduce in some cases) the bond-crossings in CG models. The first method was developed by Padding and Briels [385] for the blob model. They introduced an algorithm which prevents bond-crossings by considering a bond as an elastic band and applying the energy minimization (EM) criteria to predict the possible entanglement positions. The second method was proposed by Pan et al. [394] who added segmental repulsive forces to the force field in order to decrease the frequency of bond-crossings. Similar ideas were also put forward by Yamanoi et al. [194] and Sirk et al. [395]. While these approaches are promising, they are computationally expensive. Moreover, some parameters used in these models such as the cutoff distance of the segmental repulsions are physically ambiguous and need further explanation to avoid arbitrary choices. The third method was introduced by Nikunen et al. [396] who could prevent bond-crossings by incorporating simple topological constraints. Using this approach, Rouse as well as reptational dynamics [397] were simulated correctly for short and long chains, respectively. In spite of these attempts, there are still serious computational limitations regarding these methods which necessitate careful selection and implementation of such approaches [398].

##### High Coarse-Graining Degrees

The coarse-graining methods discussed so far often lump a few atoms up to several monomers into a single super atom. Since the polymer chain length is typically much longer than these coarse-graining limits, super coarse-graining models are necessary to approach extremely large spatial and temporal scales of polymers. In such models, an entire polymer chain is often represented by a single particle. The dynamics of polymer chains is strictly defined by the dynamics of the centers of mass of these particles and all the high-frequency motions associated with macromolecules are dropped out. Based on these ideas, a super CG model was developed by Murat and Kremer [399] in which polymer chains were replaced by soft ellipsoidal particles. The size and shape of the particles is determined based on the conformations of the underlying chains. The internal energy of a particle with a given size is characterized by the probability of occurrence of that particle. Furthermore, the density of monomers within each particle is calculated from all conformations that have the same size. The spatial overlap of the monomer density distributions of two particles defines the interaction between them. For a large number of contacting particles, the interactions between the particles forces them to adjust the equilibrium size distribution. Their simulations showed that the generic Gaussian random walk scheme appropriately defines the behavior of the chains in the melt [399]. They argue that a large number of long chains can be simulated within a reasonable computation time on a single workstation processor due to the fact that the internal degrees of freedom of the chains are severely smeared out [399]. Extensions of this method are available in which a chain of such soft particles can be considered for the simulations of high molecular weight polymers [400,401,402,403]. For instance, Zhang et al. [403] used such a strategy in combination with the mapping of the density distributions onto a lattice in the framework of MC schemes and could develop a particle-to-mesh approach for high molecular weight polymers. The authors propose that such a grid-based scheme could be a viable candidate to produce equilibrated models of long polymer chains useful in the setting of a general multiscale study [403].

**Figure 8:**
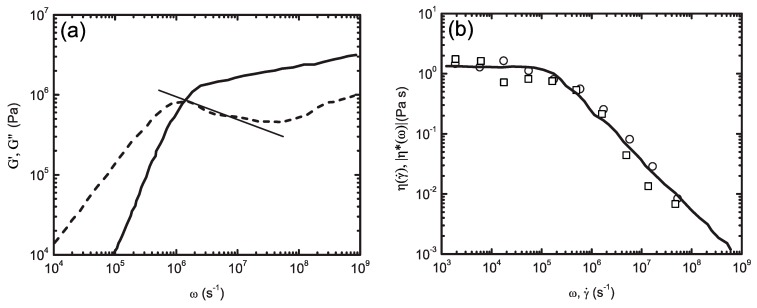
(**a**) Storage G′ and loss G″ moduli (full and dashed lines, respectively); and (**b**) the flow curve for C_800_H_1602_ melt at 450 K. Reprinted from Kindt and Briels [404] with the permission of AIP Publishing. The solid line in (**b**) is derived in equilibrium simulations using the Cox-Merz rule. The circles and squares are simulation results under shear benefitting from linear background and variable flow field methods, respectively. For further information regarding the data shown in figure refer to the cited work and the references within it.

An interesting super CG model was developed by Kindt and Briels [404] in which a single particle was ambitiously used to study the dynamics of entangled polymer chains. In this model, a set of entanglement numbers are used for each pair of particles to describe the deviation of the CG model (with the ignored degrees of freedom) from the equilibrium state. Such deviations give rise to transient forces in the system. The displacements of the particles are governed by these transient forces as well as the conservative forces derived from the potential of mean force. This deviation-displacement analysis is performed for any given configuration of the centers of mass of the polymers. Due to the core role of the transient forces in the simulation strategy, it has been called the “transient force model” [405]. The authors applied this model to a melt of C_800_H_1602_ chains at 450 K and examined radial distribution functions, dynamic structure factors, and linear and nonlinear rheological properties. In general, they could achieve good qualitative, and to a large extent quantitative, agreement with experiments and more detailed simulations. Figure 8 illustrates typical linear and nonlinear rheological properties for C_800_H_1602_ chains at 450 K calculated by Kindt and Briels [404]. The surprising observation that a single particle could capture the correct reptation behavior was qualitatively linked to the transient forces being quadratic in the deviations of entanglement numbers and thus resembling the confined motions of a chain in a tube [405]. This model has been further applied to study rheological properties of various polymer systems [406,407,408,409,410,411,412].

Based on analytical calculations through the Ornstein-Zernike equation [413], a super coarse-graining model was developed by Guenza and her co-workers [363,364,365,414,415,416,417,418,419] which does not need any further optimization against a more detained model. This model provides analytical expressions for various thermodynamic and physical quantities which are especially useful when dealing with rescaling issues. As it was noted before, once a molecule is coarse-grained its entropy as well as accessible surface to the surrounding molecules are changed. The entropy change becomes important in such super CG models in comparison with low coarse-graining degrees such as IBI. The present model provides analytic expressions for the scaling factors from each contribution as [363,365]

(51) $$s_{\mathrm{entropy}}{\;=\;R}_{g}\sqrt{\frac{{3\mathrm{MN}}_{c}}{{2k}_{B}T}},$$

(52) $$s_{\mathrm{friction}}\;=\;\frac{\xi }{{N\xi }_{m}},$$

with $s_{\mathrm{entropy}}$ and $s_{\mathrm{friction}}$ as the rescaling factors for the entropy and surface changes, respectively. Here, M is the molecular weight of the chain with radius of gyration $R_{g}$, and $N_{c}$ is the number of monomers per chain. ξ and $\xi_{m}$ are the friction coefficients of the super CG and freely-rotating chain systems, respectively.

#### 3.1.2. Reverse Mapping

While the coarse-graining procedure helps accessing longer time scales in simulations, it also removes detailed atomistic features necessary for precise evaluations of the structure. Since CG models have proven extremely useful in various simulations, such as generating equilibrated structures for further analysis and simulation runs [350,420,421,422], there is a general tendency towards employing them upon possibility. Consequently, a reverse mapping is also needed to reproduce atomistic details such as chemical characteristics from the CG model. The reverse mapping procedure is also referred to as fine-graining or backmapping in the literature [423,424].

Early attempts for reverse mapping are dated back to Tschöp et al. [425] and Kotelyanskii et al. [426]. In general, a reverse mapping operation includes (i) the reconstruction of CG particles with possible atomistic structures from a bank of templates; followed by (ii) performing EM, MD, or MC simulations to guarantee collectively and locally relaxed atomistic structures. In the first step, the fitting templates are often extracted from a preceding atomistic equilibrium simulation. The chosen template for a given CG particle should not only fit the contour of the underlying CG molecule, but also allow the best superposition for the neighborhood CG particles. In order to achieve a high backmapping efficiency, the fitting procedure is usually based only on geometrical criteria and no force and energy calculations are involved. In some cases where the CG particle represents a complex structure with bulky side groups, one must be careful to avoid interlocking of side groups [420]. In the second step, it is necessary to run post-processing calculations due to the fact that the CG force field is derived from average atomic distributions and therefore may easily lead to overlapping structures [427]. Such artefacts could happen more frequently in coarser CG models.

**Figure 9:**
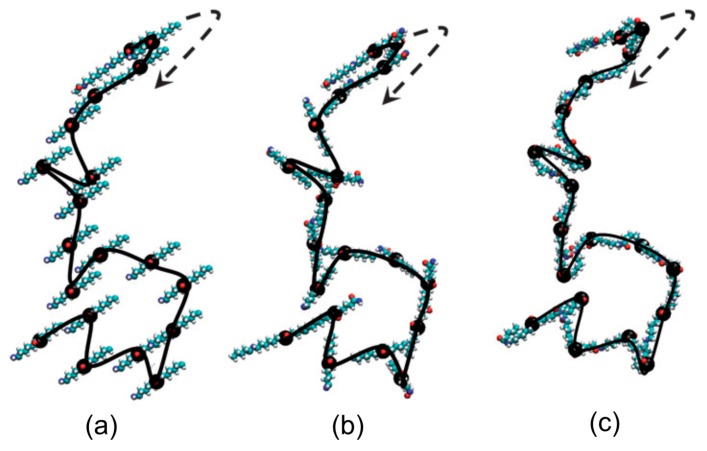
Reverse-mapping procedure for PA-66: (**a**) insertion of the atomistic segments (colored beads) on the underlying random walk chain (solid black line); (**b**) re-orientation of the atomistic segments; (**c**) final configuration of the reconstructed atomistic chain. The arrow indicates the grow direction of the chain. Reproduced from Carbone et al. [424] with permission of The Royal Society of Chemistry.

Several backmapping approaches are proposed for different polymers in the literature [420,425,428,429,430,431]. Often, when the CG model is constructed based on the atomistic simulations, the zoom-in back to the atomistic description is simply a geometrical problem [430]. However, a more sophisticated procedure must be followed in some cases where the model is significantly coarse or the CG particles include asymmetric atoms and the polymer chain shows a specific tacticity [420,431]. An example for the first case was given by Karimi-Varzaneh et al. [430] who used a simple backmapping algorithm to reinsert the atomistic details of a PA-66 in its corresponding CG model. As for the latter, Wu [431] utilized a special backmapping procedure to capture tacticity effects on the structure and dynamics of poly(methyl methacrylate) melts. Moreover, a general backmapping technique to prepare equilibrated polymer melts was proposed by Carbone et al. [424] which consists of (i) the generation of random walk chains with various Kuhn lengths; and (ii) the insertion of atoms on the underlying random walk chains. The steps of this approach for PA-66 are shown in Figure 9. The authors showed that well-equilibrated melts of PE, atactic PS and PA-66 can be achieved using this method. The structural properties of such relaxed melts were shown to be in good agreement with previous AA simulations and experimental data on short as well as long spatial ranges. Some cases with special reverse mapping algorithms are also found in literature. For instance, in order to generate realistic amorphous polymer surfaces, Handgraaf et al. [432] developed a special mapper which takes the CG structure as input and uses the MC technique to generate the atomistic structure. The mapped atomistic structure is later equilibrated by performing a short MD simulation.

**Figure 10:**
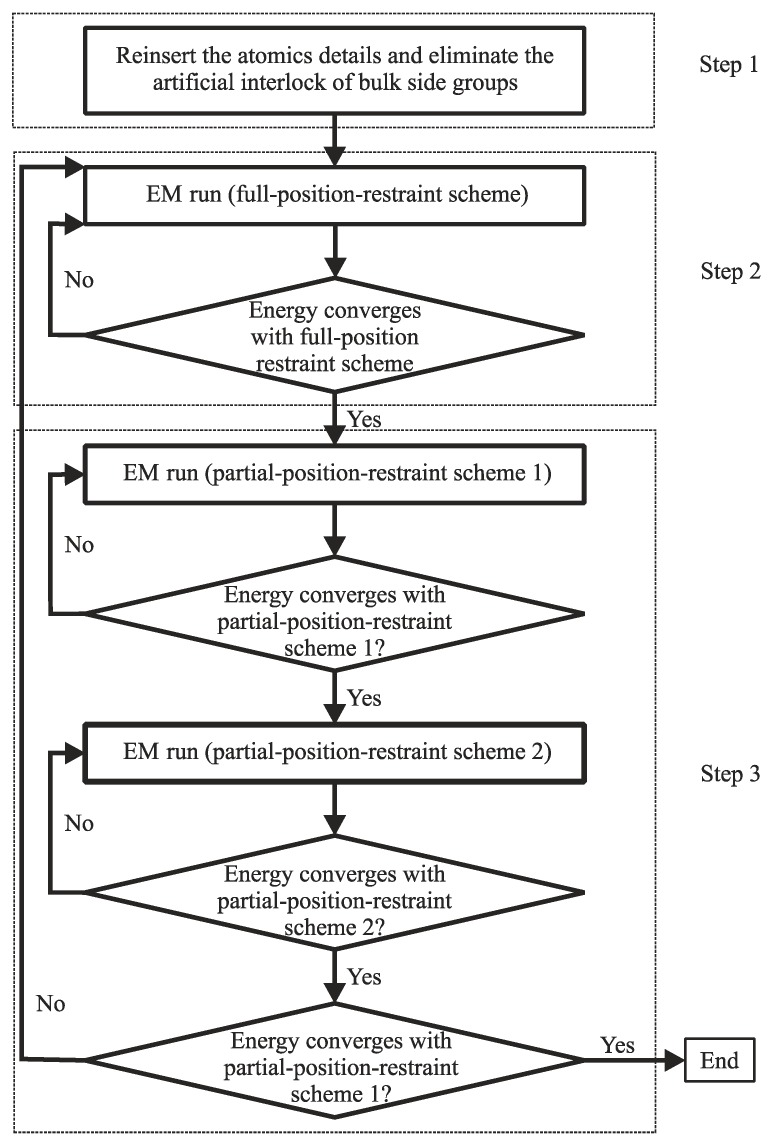
The workflow used in the backmapping procedure of nonequilibrium CG simulations as proposed by Chen et al. [423]. Notice that schemes 1 and 2 in step 3 are two variants of the main scheme in step 2 in order to minimize the isolation of segments from the rest of the chain. Reproduced from Chen et al. [423] with permission of the PCCP Owner Societies.

It should be noted here that the reverse mapping of a nonequilibrium CG system differs from an equilibrium run to some extent. Since molecular deformations are significant in the CG model due to the nonequilibrium simulations, a proper backmapping procedure should translate these deformations into the atomistic model. Furthermore, the atomistic model must also contain information about the stored deformation energy in the CG model of the polymer. Obviously, a simple backmapping cannot meet these requirements since during the post-processing step, i.e., EM or MD or MC simulations, the energetically unstable deformed structure relaxes quickly. A backmapping method was proposed by Chen et al. [423] to overcome this problem for polymer chains experiencing sheared nonequilibrium conditions. Their methodology mixes the general concepts of backmapping with the new idea of applying position restraints to preserve the deformed configurations. In order to preserve the stretched chain configuration obtained in the CG simulation, position restraints with a harmonic potential are applied to all the atoms coinciding with CG particles locations. The globally deformed structure is allowed to relax locally using a molecular mechanics approach [433]. By changing the position restraint scheme and re-optimizing the structure through an iterative procedure, it is possible to minimize the isolation of segments from the rest of the chain. The workflow of the backmapping procedure of Chen et al. [423] is illustrated in Figure 10.

Finally, the validity of a reverse-mapped atomistic structure is often tested by comparing relevant structural information simulated using atomistic models based on the reverse-mapped configurations with the original AA simulations initially used to develop the CG force field [424,430,434]. Radial distribution function of a specific chemical group, bond and angle distributions, torsion angle distribution, and the number of hydrogen bonds are mostly used for such comparisons. In some studies, the results of a reverse-mapped atomistic simulation are also directly compared with the available experimental data [424].

### 3.2. Concurrent Multiscale Approaches

**Figure 11:**
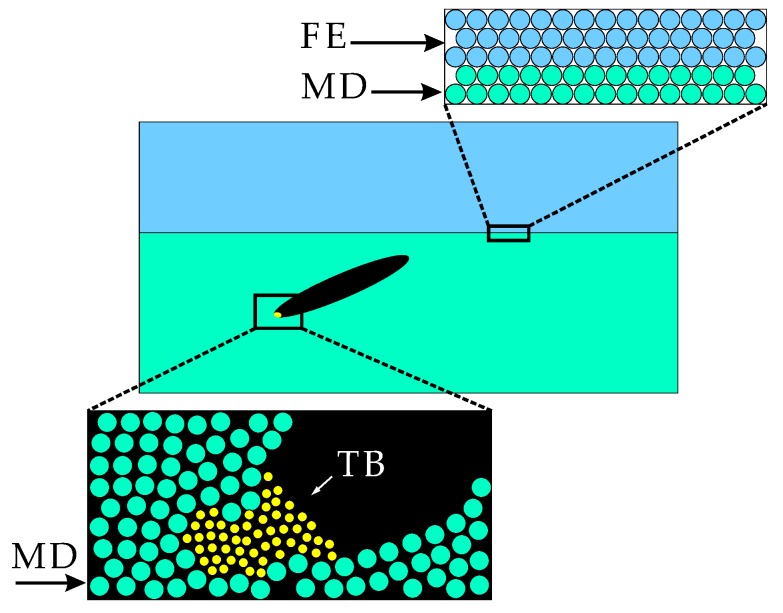
A hybrid FE/MD/TB simulation. The FE, MD, and TB approaches compute forces on particles (either FE nodes or atoms) in their respective domains of application. These forces are then incorporated to calculate the updated positions and velocities of the particles in a time-stepping algorithm.

The concurrent approaches define the system under consideration through a genius combination of several methods and solve them simultaneously instead of a hierarchical procedure as in sequential approaches. The resolution of the solution is adapted to provide an accurate representation of those regions of the system which are of particular interest. A common field of application for such strategies is the analysis of crack propagation in materials. During the crack propagation the immediate neighborhood of the crack tip, where the bond breaking is taking place, demands a higher precision in the models representation whereas a coarser model could suffice for further away from this region. An example of the concurrent methodology used in the crack analysis is shown in Figure 11. In this multiscale simulation, the concurrent approach combines tight binding (TB), MD, and FEM techniques to study crack propagation in silicon [435]. The vicinity of the crack should be simulated at a finer resolution since it exhibits significant nonlinearity. Therefore, atomistic MD method could provide a more precise representation of the crack surrounding whereas FEM can still accurately describe the rest of the system further away from the crack. In order to provide a reliable description of the underlying physics, the formation as well as the rupture of covalent bonds must be treated with quantum mechanics rather than empirical potentials. This is due to the fact that bonds are principally the sharing of valence electrons at a quantum mechanical scale [436]. Consequently, it is crucial to apply a TB modelling to a small region in the immediate vicinity of the crack tip, where bond breaking prevails during fracture, while the empirical potential description of MD is adequate further away from this region.

The concurrent approach is best suitable for the systems with an inherent multiscale character. In such systems, the behavior at each scale depends strongly on the phenomena at other scales. Moreover, this approach can be of a more general nature due to the fact that it does not often rely on any system-specific assumptions such as a particular coarse-graining model. Therefore, a well-defined concurrent model can be applied to many different systems within the limits of common phenomena involved as long as it incorporates all the relevant features at each level. In contrast to sequential methods, concurrent models are not usually constructed based on a detailed prior knowledge of the physical quantities and processes involved. As a result, such models are particularly useful when dealing with new emerging problems about which little is known, for instance, at the atomistic level and its connection to larger scales. However, the coupling between the different regions treated by different methods is a critical challenge remaining in the core of concurrent approaches. A successful multiscale model seeks a smooth coupling between these regions. Here, we address some of the concepts and strategies developed in the concurrent framework.

#### 3.2.1. The Concept of Handshaking

In concurrent simulations, often two distinct domains with different scales are linked together benefitting from a region called the “handshake” region. The handshake region generally bridges the atomistic and continuum domains of the multiscale model [437,438]. However, there are studies where it has been used to link quantum mechanical TB calculations to atomistic domains [438,439], or atomistic MD models to their equivalent CG descriptions [437].

The handshake region transfers information from one domain to the other and thus provides the possibility to overlap, usually, atomistic and continuum domains. This overlap is defined with a field variable, often the potential energy, taking a weighted form of the magnitude of the same variable in each domain. The weighting is usually in the form of a function which decreases monotonically from one to zero in the overlap. As a result, the control variable has its corresponding values in each domain with a gradual transition between the domains. The form of the weighting function is not determined by the formulation and is arbitrary. Consequently, the modelling quality of the handshake region is strongly dependent on a smooth and gradual shift of control variables from one domain to the other domain. In the handshake algorithm, it is assumed that the properties of each domain are independent from one another. Due to this assumption, one has to be concerned particularly whether or not the material properties of both domains are truly equivalent. In addition, physical complications in the handshake region might necessitate more complex algorithms to obtain a precise representation of it. For instance, nodal displacements of the continuum domain should be influenced by the displacements of molecules inside the neighboring atomistic domain if the node and the molecules are within the cutoff distance of the molecular interactions.

The handshaking approach has been applied to combine TB/MD/FEM in order to study crack propagation and crystal impact in silicon [438,439]. A combination of TB/MD/FEM has also been utilized in a handshaking framework to characterize submicron micro-electro-mechanical systems by Rudd et al. [437]. Based on the works of Abraham et al. [439,440] the unifying theme for such a multiscale model is the total Hamiltonian $H_{tot}$ defined throughout the entire system. This Hamiltonian is a function of the atomic positions $r_{j}$ and their velocities $v_{j}$ in the TB and MD regions for all j atoms, and the displacements $u_{\alpha }$ and their time rates of change ${\overset{\cdot }{u}}_{\alpha }$ in the finite element (FE) regions for all α nodes. Within this scheme, the Hamiltonian is divided into FE, MD, TB and handshaking contributions from FE/MD and MD/TB during the domain decomposition. It is assumed that the atomic and nodal movements are not necessarily exclusive to a single domain, but their interactions are. In this way, $H_{tot}$ may be written as

(53) $$H_{tot}=H_{FE}(u_{\alpha },{\dot{u}}_{\alpha })+H_{FE/MD}\left(r_{j}{,v}_{j}u_{\alpha },{\dot{u}}_{\alpha }\right)\;{+\;H}_{MD}\left(r_{j}{,v}_{j}\right){\;+\;H}_{MD/TB}\left(r_{j}{,v}_{j}\right){\;+\;H}_{TB}\left(r_{j}{,v}_{j}\right)\;,$$

with the Hamiltonian of different contributions depicted with appropriate indices. Rudd et al. [437] explain that the FE/MD as well as MD/TB handshakes must successfully address the fundamental issues of (i) matching the degrees of freedom and (ii) defining consistent forces at the corresponding interfaces. Despite this similarity, it should be emphasized that each handshake obliges a somewhat different approach in order to answer the requirements. This is due to the fact that the MD/TB handshake occurs across an interface of atoms whereas the interface at the FE/MD handshake is between planes of atoms [437]. Appropriate derivatives of this Hamiltonian function can be used to define the equations of motion in a standard Euler-Lagrange routine. The time evolution of all the variables can then proceed to the next step using the same integrator. The interested reader is referred to the work of Rudd et al. [437] for further information.

#### 3.2.2. Linking Atomistic and Continuum Models

It is frequently observed in large-scale atomistic simulations that only a small subset of atoms actively participate in the evolving phenomenon. This allows for the majority of atoms to be effectively represented by continuum models. Hence, a considerable reduction of computation and storage resources is guaranteed if only novel multiscale approaches could reduce the number of degrees of freedom in atomistic simulations. There is a tremendous amount of concurrent multiscale modelling methods developed in the last twenty years which couple atomistic simulations such as MD with continuum simulations such as FEM [441,442]. The idea behind these methods, not unlike all multiscale strategies, is to focus the available computation power where it is needed by applying atomistic simulations, whereas an approximate solution is provided for the rest of the system by continuum simulations. Therefore, both atomistic details as well as the macroscopic properties of materials can be obtained simultaneously from these simulations. Such models are mostly designed for crystalline materials such as metals or carbon nanomaterials. Unfortunately, their application in polymeric materials is still limited, possibly due to the unfamiliarity of these models to polymer researchers. Although some authors have referred to such methods in recent reports on polymer simulations [32,299], the fundamentals of the methods are not brought to discussion. We believe that the basic ideas of these methods can be extended to study polymeric materials. Here a brief description of these methods is provided with emphasis on the fundamentals. At the end of this section, several studies in polymeric systems are listed where such methods or a modified version of them are incorporated to address the phenomena. It is our hope that it will help guide future improvements.

**Figure 12:**
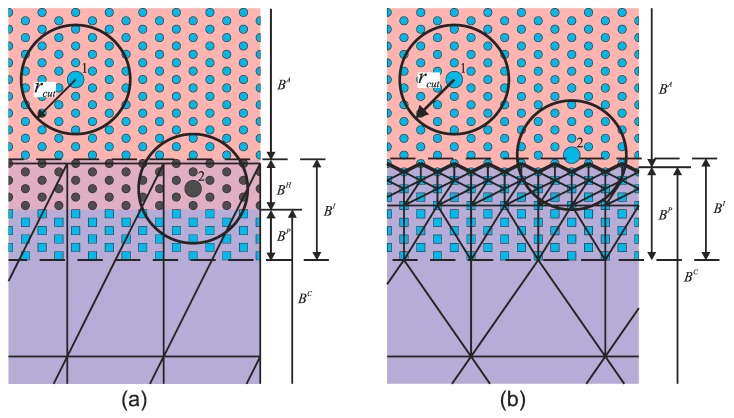
Schematic representation of generic interfaces used in coupled atomistic/continuum simulations: (**a**) with the handshake region; and (**b**) without the handshake region. Atom 1 does not influence the continuum directly (while atom 2 does) because of the finite cutoff length. Padding, handshake, and regular atoms are depicted by blue squares, black circles, and blue circles, respectively.

Certain categories of problems such as fracture and nanoindentation possess the characteristics of localized deformation where it is possible to address the system by a dual-domain or partitioned-domain approach; one with an atomistic description $B^{A}$, and the other with continuum approximation $B^{C}$. The two domains are linked by an interfacial region $B^{I}$ across which compatibility and equilibrium are enforced. An important distinction among various methods is the way they treat the interfacial region. Most methods follow one of the strategies demonstrated in Figure 12. The interfacial region is shown by the dashed lines. In part (a) of the figure, $B^{I}$ has been further subdivided into two parts: (i) the handshake region $B^{H}$, and (ii) the padding region $B^{P}$. As explained before, the handshake region provides a mixing between the two scales. The padding region is continuum in nature and provides the boundary conditions to the atoms in $B^{A}$ and $B^{H}$ with a certain range of atomistic interactions, $r_{cut}$. The thickness of this region depends on $r_{cut}$ and the motions of atoms in $B^{P}$ are calculated, in different ways for different methods, based on the continuum displacement fields at the positions of the padding atoms. It is also possible to eliminate the handshake region as shown in part (b) of Figure 12. Models that do not use a handshake region mostly incorporate a direct atom-node correspondence at the edge of the FE region to impose the displacement compatibility across the interface. This necessitates that the mesh is refined down to the atomic scale on the continuum side of the interface and hence introduces difficulties in mesh generation.

The coupling between the $B^{A}$ and $B^{C}$ domains necessitates compatibility conditions in each direction. Therefore, the displacements of atoms in $B^{P}$ must be determined from the nodal displacements in $B^{C}$. Moreover, the displacement boundary conditions need to be defined for the $B^{C}$ nodes at the edge of the mesh closest to the $B^{A}$. The compatibility criteria can be either strong or weak. The strong compatibility is when the padding atoms move in the same as the finite elements in which they reside. In this type of compatibility, subsets of nodes are defined that coincide with some of the atoms in $B^{A}$. The displacement boundary condition is therefore imposed on $B^{C}$ with the motion of the overlaying atoms from $B^{A}$. The weak compatibility, on the other hand, utilizes some sort of an averaging or penalty method to enforce the displacement boundary conditions. Strong compatibility introduces complications in mesh definition near the interface while it also yields relatively more accurate results [442].

The simulation algorithm often finds the equilibrium by either minimizing an energy functional or driving the set of forces on all degrees of freedom to zero. Consequently, there are two major categories of the governing formulation i.e., the energy-based and the force-based approaches. The major drawback of the energy-based method is that it is extremely complicated to remove the non-physical artifacts of the coupled energy functional. This problem, often referred to as the “ghost forces”, stems from trying to combine two energy functionals from different models into a single coupled energy expression [442,443,444]. The force-based approaches, on the other hand, have no well-defined total energy functional and are considered to be non-conservative in general. These approaches can be numerically slow and unstable and could converge to unstable equilibrium states. However, force-based methods can eliminate the ghost forces due to access to the direct definition of the forces.

Several methods are proposed in literature to correct the ghost forces artifact in energy-based models. These methods take various actions in order to eliminate or at least mitigate for ghost forces [445,446,447,448,449]. One such approach with general characteristics is the deadload ghost force correction [444]. In this approach, the ghost forces are explicitly computed and the negative of these forces are added as deadloads to the affected atoms or nodes. The deadload ghost force correction has shown great promise in some static simulations [442]. However, the deadload correction is only an approximation for the simulations where ghost forces change during the calculation progress.

The general algorithm for energy-based methods defines the total potential energy of the entire system $U^{tot}$ as the sum of the potential energies of the atomistic $U^{A}$, continuum $U^{C}$ and handshake $U^{H}$ regions, as

(54) $$U^{tot}{\;=\;U}^{A}{\;+\;U}^{C}{\;+\;U}^{H},$$

and minimizes it to reach equilibrium. These energies are described by [442]

(55) $$U^{A}\;=\;\sum_{\alpha \in B^{A}}E_{\alpha }\;-\;\sum_{\alpha \in B^{A}}f_{\alpha }\cdot u_{\alpha },$$

(56) $$U^{C}\;=\;\sum_{e=1}^{N_{e}}\sum_{q=1}^{N_{q}}\omega^{q}V_{e}W(∆(r_{e}^{q}))\;-\;{\bar{f}}^{T}u,$$

(57) $$U^{H}\;\approx \;\sum_{\alpha \in B^{H}}(1-\Theta \left(r_{\alpha }\right){)E}_{\alpha }+\sum_{e\in B^{H}}\Theta \left(r_{e}^{\mathrm{cent}}\right)W\left(\Delta \left(r_{e}^{\mathrm{cent}}\right)\right),$$

where the energy, spatial coordinates, displacement and applied forces of atom α are shown by $E_{\alpha }$, $r_{\alpha }$, $u_{\alpha }$, and $f_{\alpha }$, respectively. $N_{e}$ is the number of elements, $V_{e}$ is the volume of element e, $N_{q}$ is the number of quadrature points in the numerical integration, $r_{e}^{q}$ is the position of quadrature point q of element e in the reference configuration, and $\omega^{q}$ is the associated Gauss quadrature weights. $\bar{f}$ and u are the vector of applied forces and nodal displacements in the FE region, respectively. W is a function of the deformation gradient Δ. $r_{e}^{\mathrm{cent}}$ is the coordinates of the Gauss point in element e which is taken at the centroid of the triangular elements in this specific case shown in Figure 12. One should notice that the energy of the continuum region is approximated due to the fact that a continuous integral has been replaced by a discrete numerical method. Consequently, the handshake region is also approximated since it also uses such a numerical approach for the continuum energy contribution. In the energy equation for the handshake region, both the continuum and atomistic energies are used in a weighted fashion according to a function Θ which varies linearly from one at the edge of $B^{H}$ closest to the continuum region, to zero at the edge closest to the atomistic region. Indeed, for methods with no handshake region, $U^{H}$ is taken zero and only the continuum and atomistic regions contribute to $U^{tot}$. Moreover, one should note that the padding atoms have no contribution to the formulation of the potential energy. Therefore, these atoms only provide an appropriate boundary condition for the atoms in $B^{A}$.

The force-based methods are based on two independent potential energy functionals. The first one calculates an energy functional $U^{\mathrm{atom}}$ assuming the entire system is modelled using atoms. The second energy functional $U^{\mathrm{FE}}$ on the other hand, provides a description of the system if it was modelled entirely in a FEM framework. The forces for all α atoms, $f_{\alpha }$, and all i nodes, $f_{i}$, are simply found by differentiating the corresponding energies with respect to the atomic or nodal displacements, $u_{\alpha }$ and $u_{i}$ respectively, as

(58) $$f_{\alpha }\;=\;\frac{\partial U^{\mathrm{atom}}}{\partial u_{\alpha }},$$

(59) $$f_{i}\;=\;\frac{\partial U^{\mathrm{FE}}}{\partial u_{i}}.$$

It is important to note that the difference between energy-based and force-based methods stems from the fact that in the second approach one does not attempt to minimize the combined energy functional. In the following, some relevant approaches which are used to link atomistic and continuum models are discussed.

##### Quasicontinuum Approach

Quasicontinuum (QC) method is a particularly interesting approach by Tadmor et al. [450,451,452] which seamlessly couples the atomistic and continuum realms. In QC approach, the atomistic description of the system is systematically coarsened by the introduction of kinematic constraints designed carefully so that the full atomistic resolution is preserved where required, for instance in the vicinity of large deformations, and to treat collectively large numbers of atoms in regions further away. QC was firstly developed to investigate defects in solids considering the interaction of dislocations [444,450,451,453,454,455,456]. However, it has also found applications in fracture and crack mechanics [457,458], and nanoindentation [459].

**Figure 13:**
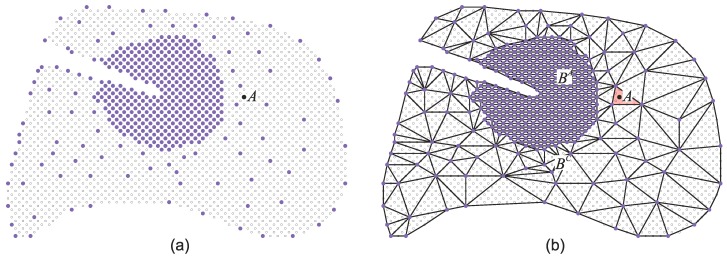
For an irregular domain which includes a crack, part (**a**) shows the representative atoms near the crack tip; Part (**b**) demonstrates the domain meshed by linear triangular elements. The density of representative atoms is adjusted to correspond to the variation in the deformation gradient. In order to calculate the displacement of atom *A* in part (**b**), one can use a linear interpolation of the displacements of the three representative atoms which form the highlighted element.

In QC method, there is no handshake region. Since there is no separation of the domains in QC, there are no needs for separate sets of material data in this multiscale approach. This is a significant advantage of QC. The calculation domain is partitioned into non-overlapping cells similar to the FEM. These cells then cover the constituting molecules of the material while their vertices coincide with some representative atoms from the molecules. The local density of such representative atoms is larger in regions with high deformations compared with the regions experiencing low deformations. Figure 13 shows an example for the selection of representative atoms in the vicinity of a crack. QC takes the degrees of freedom in a cell the same as the degrees of freedom of the representative atoms of that cell. In addition, the movement of molecules is usually calculated from the representative atoms utilizing interpolation functions. QC also approximates the average energy of a cell from its representative atoms. The method eventually looks for the arrangement of representative atoms which minimizes the potential energy of the domain.

Variants of the QC model have been developed and applied in different situations [450,451,460,461]. In general, the QC approach includes three major blocks: (i) the constrained minimization of the atomistic energy of the system; (ii) the computation of the effective equilibrium equations based on appropriate summation rules; and (iii) the design of the computational mesh representing the structure of the system based on proper adaptation criteria. The QC model initially provides a full atomistic description of the system which is later scaled down to a subset of representative atoms. The positions of the remaining atoms are obtained by piecewise linear interpolations of the representative atoms. Afterwards, the effective equilibrium equations are obtained by minimizing the potential energy of the system based on the scaled-down configuration space. A precise evaluation of the total energy of the system $E^{tot}$ is often performed over the full collection of atoms as

(60) $$E^{tot}\;=\;\sum_{i=1}^{N}E_{i},$$

in which N is the total number of atoms, and $E_{i}$ is the energy of the ith atom at its corresponding position in the system. This comprehensive formula is approximated in QC models benefitting from the concept of representative atoms with

(61) $$E^{tot}\;\approx \;\sum_{i=1}^{N_{r}}\omega_{i}\bar{E_{i}},$$

where $\omega_{i}$ and $\bar{E_{i}}$ are the quadrature weight which shows the number of the atoms that a given representative atom stands for in the definition of the total energy, and the energy of the ith representative atom, respectively. Here, the summation is only performed over $N_{r}$ representative atoms and thus the calculation effort is reduced. The representative atoms are usually adaptively selected so that an accurate description of the critical positions with larger deformation fields is obtained. QC approach often incorporates FEM to determine the displacement fields and combines it with an atomistic technique which is used to determine the energy of a given displacement field. One can compare it with the standard FEM in which a constitutive law is coupled with it through a phenomenological model.

The concepts of QC could be extended to include a coupling between atomistic calculations and QM as well. Such an strategy was initially introduced to study fracture in silicon and the method was named coupling of length scales (CLS) [437,439,440]. There are small differences between QC and CLS. Initially CLS method used a small strain approximation to describe the continuum region rather than the Cauchy-Born rule used in QC [442,462]. However, conceptually the methods are similar since the original CLS approach could be generalized to provide a nonlinear Cauchy-Born description for the continuum region. Furthermore, minor differences between the methods exist in the way they treat the interface. Still, these differences are believed to have slight influences on the error and rate of convergence [442,463].

QC suffers from the ghost forces like any other energy-based method. An idea to reduce these forces was initially put forward by introducing a handshake region to the QC models. This idea along with minor changes in the manipulation of forces at the interface constructed the bridging domain method (BDM) [464]. At the interface, BDM uses weak compatibility which eliminates the need for one-to-one correspondence between atoms and nodes. This weak compatibility imposes some loss of accuracy on BDM. Another approach to correct for ghost forces is the iterative minimization of two energy functionals used in composite grid atomistic/continuum method (CACM) [465]. CACM is a highly modular method with weak compatibility and no handshake region. It provides the possibility to separately solve energy functionals of different regions. However, this could lead to longer computation times especially for nonlinear problems.

##### Coarse-Grained Molecular Dynamics

Coarse-grained molecular dynamics (CGMD) was originally developed to model the nano-electro-mechanical systems (NEMS) [437,452,466]. In this technique, conventional MD is coupled with a CG description of the system. The CG regions are modeled on a mesh in a formulation that generalizes conventional FEM of continuum elasticity. The significant aspect of CGMD is that it is derived solely from the MD model and has no continuum parameters. In other words, this method is notably different from the other coupled atomistic/continuum methods presented in this manuscript in the way that it constructs the continuum model only based on the atomistic information. As a result, it offers a smooth coupling and provides control of errors that arise at the coupling between the atomistic and CG regions. A more general version for the dynamics of CGMD is also proposed by Curtarolo and Ceder [467].

In CGMD the domain is partitioned into cells with variable sizes. This provides the possibility to assign a mesh node to each atom in important positions whereas in other regions the cells could contain several atoms and the nodes are not necessarily coincident with atoms. CGMD follows a detailed statistical coarse-graining prescription which particularly results in scale-dependent constitutive equations for different regions of the domain [466]. In CGMD, the CG mesh is refined to the atomic scale where it joins with the MD lattice. This refined mesh with no handshake region as well as the fact that CGMD adopts an effective field model suggests a strong resemblance to QC. In addition to the point made earlier on the use of atomistic constitutive equations in CGMD, this method is also designed for finite-temperature simulations. On the contrary, the classic QC is mainly applicable to zero-temperature simulations. It is interesting to note that according to Rudd and Broughton [466] the classic QC is closely related to the zero-temperature rigid approximation of CGMD. It should be noted that finite-temperature versions of QC are developed in recent years [468,469,470]. These methods often benefit from coarse-graining concepts similar to CGMD. Finally, CGMD is free from the ghost forces which is a desirable feature missing in QC.

##### Finite-Element/Atomistic Method

The finite-element/atomistic (FEAt) method is a force-based method first introduced by Kohlhoff et al. [471]. FEAt uses no handshake region and strong compatibility is enforced between the domains. To compensate for the absence of the handshake region, FEAt incorporates a nonlocal elasticity formulation in the finite elements and mitigates the abrupt transition from $B^{C}$ to $B^{A}$. In general, the forces on every atom α in $B^{A}$ and $B^{P}$ are calculated independently from $B^{C}$, from the derivative with respect to atom positions of an energy functional $U^{A\cup P}$ of the form

(62) $$U^{A\cup P}\;=\;\sum_{\alpha \in \;\left\{B^{A}\cup B^{P}\right\}}E_{\alpha }\;-\;\sum_{\alpha \in \;\left\{B^{A}\cup B^{P}\right\}}f_{\alpha }\cdot u_{\alpha }.$$

This energy functional looks very similar to the one used in energy-based methods, but it is fundamentally different since it also contains the padding atoms. The energy functional of the continuum domain is similar to the energy functional of the energy-based methods described in Equation (56). The forces on the nodes are therefore simply obtained from its derivative with respect to nodal displacements. Based on these forces, the atoms and nodes are moved and the forces are re-calculated for the new atom and node positions.

Some variations to FEAt are found in the literature. In the presence of dislocations in the continuum, one can use discrete dislocation methods in the description of the continuum region. The resulting continuum region could be coupled with the atomistic region in a force-based algorithm just like FEAt to yield coupled atomistic and discrete dislocation (CADD) approach [472,473]. In order to remove the strong compatibility from FEAt and CADD, the hybrid simulation method (HSM) uses the same approach as BDM by including a handshake region in the system [474]. A variation of HSM is the concurrent atomistic/continuum (AtC) method in which a blending of forces is performed at the interface [443,475,476].

##### Bridging Scale Method

**Figure 14:**
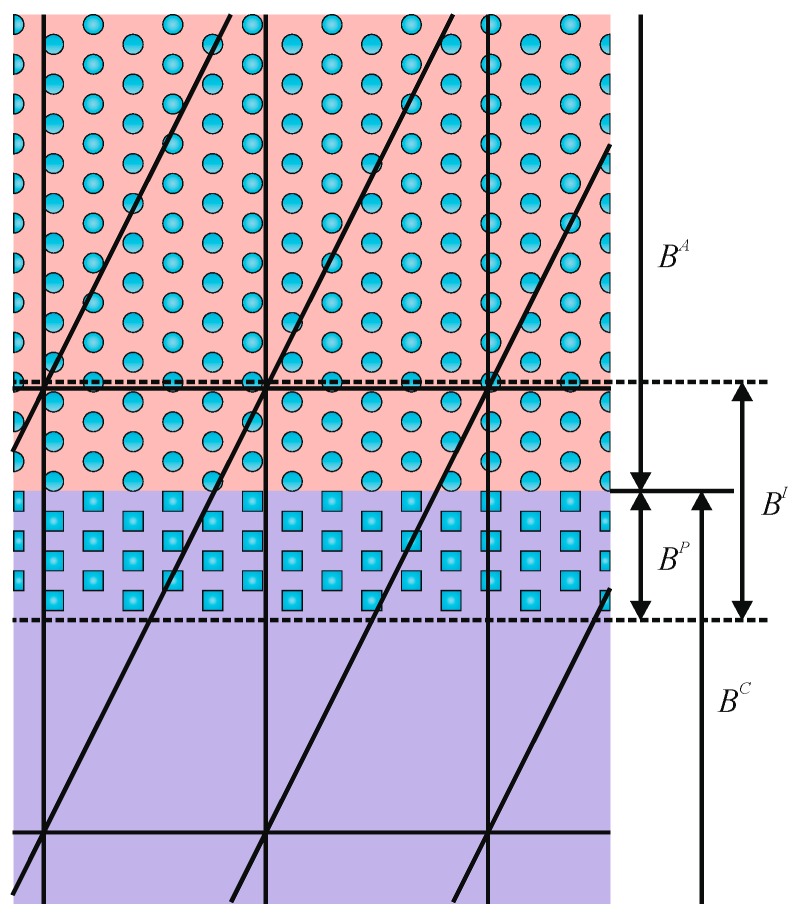
The BSM interfacial region. The interface has no handshake region and the finite elements cover the entire body which allows to store the coarse scale displacement field.

The bridging scale method (BSM) is an energy-based technique with no handshake region. In this method, the FE mesh exists throughout the entire domain in order to store a part of the final solution, see Figure 14. The central idea behind BSM is derived from classical works in decomposing a complete solution of the total displacement field into fine and coarse scales and solving for the fine scale only in regions that require it [477,478,479]. The coarse scale solution is that part of the solution which is normally represented by a set of FE shape functions. The fine scale solution on the other hand, is defined as the part of the solution whose projection onto the coarse scale is zero.

In BSM framework, the coarse scale solution $\bar{\gamma }r_{\alpha }$ is taken to be a function of the initial positions of the atoms $r_{\alpha }$ and is defined by

(63) $$\bar{\gamma }r_{\alpha }=\sum_{i}\varsigma_{i}^{\alpha }u_{i},$$

where $\varsigma_{i}^{\alpha }$ is the shape function of node i evaluated at point $r_{\alpha }$, and $u_{i}$ is the FE nodal displacement associated with node i. Using a mass-weighted least-squares fitting of the coarse scale solution to the total solution, Park and Liu [480] showed that the fine scale solution γ′ can be defined based on a projection matrix P as

(64) γ′ = γ − Pγ.

Here, γ is the exact solution determined from an underlying atomistic technique such as MD. Therefore, the total solution can be found by summing up both fine and coarse scale contributions. Such an approach is sometimes referred to as the projection method in the literature due to the fact that atomistic and continuum regions are coupled by projecting a fine scale solution onto a finite dimensional solution space [481].

##### Applications in Polymeric Materials

In this part of the paper, we give several examples for the applications of coupled atomistic/continuum models in polymeric systems. In the studies outlined here, one can find applications of the methods explained so far; either it is directly used, or a modified version is developed to capture the correct physics involved, or a concept is borrowed to propose new models for polymers. The reader should note that our goal is not to provide a comprehensive list here but merely to raise attention towards the opportunities. We hope that the polymer researcher finds it useful in order to navigate through these multiscale approaches and further develop new strategies for one’s own problem.

Generally, it is more difficult to model polymers than crystalline materials due to their amorphous nature. A methodology to solve this problem was formulated by Theodorou and Suter [482,483] in which a parent chain of atoms is attached to an Amorphous Cell (AC). The AC then experiences deformations while periodic boundary conditions are applied to all sides. Tan et al. [481] incorporated the concept of AC and developed it based on the adaptive scaling resolution ideas similar to CGMD and introduced the Pseudo Amorphous Cell (PAC) multiscale approach for amorphous polymers. PAC algorithm includes: (i) generating a configuration of polymer chains in the domain; (ii) applying linear molecular mechanics for regions with small deformations; (iii) reducing the degrees of freedom in such regions; and (iv) coupling of linear and nonlinear molecular mechanics equations. In their method, the regions with large deformations are represented with nonlinear molecular mechanics and thus provide a finer solution. The authors showed that PAC can successfully simulate the nanoindentation of amorphous polymers and the indentation force was predicted with a good precision comparable to a full molecular mechanics simulation [481]. Later Su et al. [484] applied the PAC approach to correlate the movements of atoms of an amorphous material within a representative volume element (RVE) to the its overall deformation.

The ground idea of projection methods was first introduced in details by Hughes et al. [477] as the variational multiscale methods (VMS) which allows a complete model to be described by orthogonal subscale models. Utilizing this property, Codina [485] presented a method to deal with numerical instability of the Stokes problem due to the incompressibility constraint and convection. He proposed using orthogonal subscales in FEM through the pressure gradient projection. This approach has been developed recently by Castillo and Codina [486,487] to present stabilized VMS formulations to solve the quiescent three-field incompressible flow problems of viscoelastic fluids as well as fluids with nonlinear viscosity. The authors were able to successfully capture the distributions of streamlines in a sudden contraction flow for an Oldroyd-B fluid at Re of 1 at various Weissenberg numbers (We). It was observed that the size of the vortex appearing in the bottom corner decreases as We increases.

In a recent MD study of brittle fracture in epoxy-based thermoset polymers under mechanical loading, Koo et al. [488] introduced an EM step into the virtual deformation test to maintain the system temperature at zero. They stated in the paper that this idea was borrowed from QC which bridges atomistic scale to continuum scale by decoupling temperature effects. The possibilities of incorporating multiscale approaches to connect MD and FEM such as QC, in investigations of structure at epoxy-silica interface are also emphasized by Büyüköztürk et al. [489].

Jo and Yang [490] utilized an atomistic/continuum model to predict the mechanical properties of semicrystalline poly(trimethylene terephthalate) (PTT). Their approach includes an EM process similar to energy-based methods. The semicrystalline PTT includes an amorphous matrix represented as a continuum, and the crystalline phase represented by a spherical inclusion modelled in atomistic detail. The degree of crystallinity of PTT is altered by changing the volume fraction of an inclusion.

In order to model the compressive behavior of carbon nanotube PNCs, Li and Chou [491,492] developed a multiscale strategy in which the nanotube is modelled at the atomistic scale, and the matrix deformation is analyzed by the continuum FEM. Their methodology is similar to other atomistic/continuum coupling themes except for the fact that they adopt a so-called truss rod model to correctly represent van der Waals interactions at the interface. The multiscale scheme developed by Li and Chou was later incorporated by Montazeri and Naghdabadi [493] to study the stability of carbon nanotube PNCs with a viscoelastic matrix. They coupled molecular structural mechanics to FEM and simulated the buckling behavior of the system.

A multiscale simulation strategy was proposed by De et al. [494] to determine the mesoscopic velocity development in polymer fluids with large stress relaxation times. The incorporation of a constitutive viscosity equation is not sufficient in such systems to produce the correct rheology. The authors introduced a scale bridging concept in which small parts of the system were simulated with MD. These parts could communicate with each other through a continuum approach. During the passing of information, the continuum approach provides precise means of interpolating between these points. They described the coupling of atomistic and continuum regions in a Lagrangian framework so that the memory effects are included in the calculations.

### 3.3. Adaptive Resolution Simulations

It was already discussed in the concurrent multiscale approaches that there is a category of systems in which the phenomenon of interest is focused in a subregion of the entire domain. Consequently, it would be computationally efficient if the irrelevant AA representation of molecules far from this subregion were replaced with an alternative less expensive model. However, the common limitation in all concurrent methods (introduced so far) is that particle exchange is not allowed in the fixed regions of the system treated at different resolutions. The relatively new class of multiscale simulation approaches, i.e., the adaptive resolution simulations, provides this possibility. Several papers have been devoted to address different aspects of these methods in recent years showing their increasing popularity [337,495,496,497,498]. It should be noted that these methods can be principally considered to be concurrent since they often couple the simultaneous run of two techniques with different levels of resolution using a transition region. Furthermore, the transition region usually uses an either force or energy interpolation criterion to link different resolutions somewhat similar to the concurrent methods. However, in adaptive resolution simulations, an atom or a molecule is free to smoothly switch its resolution within the same simulation run depending on its spatial coordinates. Therefore, it allows for an adaptive modification of the resolution within the coexisting models which promotes the accuracy where needed and provides the required precision. In concurrent approaches, on the other hand, different scales are coupled often by a step-wise transfer of information between different methods, for instance we refer to Youn Park et al. [499]. Therefore, some authors introduce adaptive resolution simulations as a separate class of multiscale approaches to emphasize these different aspects [32]. Here, we also follow this notion.

**Figure 15:**
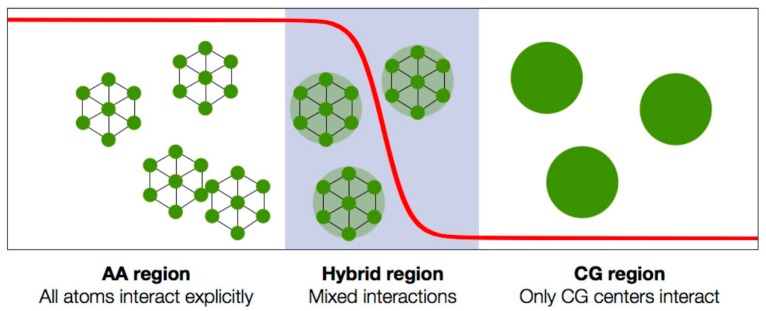
Representation of an adaptive resolution simulation in which a high-resolution region (AA region) is coupled to a low-resolution region (CG region). In the AA region, the structure of the molecules are described in their full atomistic details. In the CG region, however, a simpler representation of the structure and interactions of the molecules are utilized. A transition region is used to connect these regions. The novelty as well as difficulty of adaptive resolution schemes depends strongly on the properties of the transition region, i.e., the way molecules change their resolution. Reprinted from Potestio et al. [337] under the terms of the Creative Commons Attribution License.

**Figure 16:**
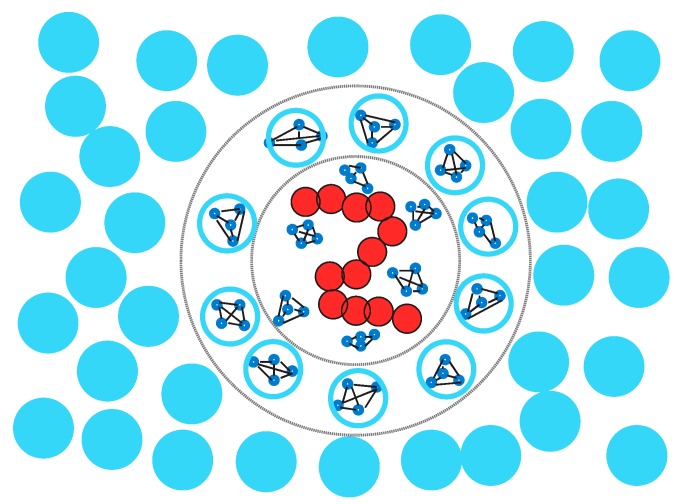
A schematic representation of a generic polymer solution. The structural resolution of the solvent molecules adaptively change based on their distance from the center of the mass of the polymer chain. The polymer beads are represented smaller than the solvent molecules to preserve clarity. Reprinted from Praprotnik et al. [500] with the permission of AIP Publishing.

The adaptive resolution simulations often divide a domain into an AA and a CG region and link them using a transition region, see Figure 15, hence are sometimes referred to as the double-resolution simulation methods. Examples for the appropriate systems to investigate with such a strategy include the studies of macromolecules embedded in a solvent (see Figure 16) [500], and liquids near surfaces [501]. The transition region provides the basis for a smooth interpolation from a certain structural representation of a molecule to another depending on the properties that have to be preserved in the CG region. A complete methodology should address the interactions between the atoms or molecules in different domains as well as the property change in crossing the transition region. Moreover, it is central to adaptive resolution simulations that the molecules should be able to diffuse freely between different regions of the simulation box. Other constraints could include thermal equilibrium and uniform density profile across the entire domain which along with certain region-specific properties lead to a formulation of an adaptive resolution scheme.

#### 3.3.1. The Adaptive Resolution Scheme

The Adaptive Resolution Scheme (AdResS) was developed by Kremer and co-workers [500,502,503,504,505,506,507] to simulate systems in which an AA and a CG model are incorporated to model different subregions of the simulation domain at the same time. The atoms and molecules are allowed to diffuse freely from one region to the other using a smooth transition region which links the subregions. AdResS is principally based on the assumption that Newton’s third law should be satisfied the entire simulation box. Additionally, the method assumes that a molecule in the CG subregion contains no information about its atomistic details and interacts with other molecules, either in AA or CG regions, only via its center of mass. An interpolation scheme for the force field across the domain defining the force $f_{\alpha \beta }$ acting between molecules α and β can be formulated considering the aforementioned assumptions as

(65) $$f_{\alpha \beta }\;=\;\psi \left(R_{\alpha }\right){\;\psi (R}_{\beta }{)\;f}_{\alpha \beta }^{\mathrm{AA}}{\;+\;(1\;-\;\psi (R}_{\alpha }{)\;\psi (R}_{\beta }{))\;f}_{\alpha \beta }^{\mathrm{CG}},$$

where $R_{\alpha }$ and $R_{\beta }$ are the center of mass coordinates of molecules α and β, respectively. $f_{\alpha \beta }^{\mathrm{AA}}$ and $f_{\alpha \beta }^{\mathrm{CG}}$ are the atomistic and CG forces acting on molecule α due to the interaction with molecule β, respectively. Here, ψ is a spatial interpolation function that goes from 1 in the AA region to 0 in the CG region smoothly. In the transition region, atomistic details are explicitly integrated and the CG force is computed between the centers of mass of the molecules and then redistributed to the atoms weighted by the ratio of the atom’s mass to the mass of molecule [508]. In the CG region, the CG force is directly applied to the center of mass coordinates of the molecules and there is no need to conserve the molecules internal structure. When a molecule enters the CG region its atomistic details are removed and reintroduced again, through some sort of reservoir of equilibrated atomistic structures, as soon as it approaches the transition region.

The central requirement of satisfying Newton’s third law in AdResS is demonstrated to rule out any form of potential energy interpolation and vice versa [509]. Consequently, energy-conserving simulations in the microcanonical ensemble cannot be performed using AdResS. Due to the non-conservative nature of the forces in the transition region, molecules receive an unreal excess energy when crossing this region. This energy can be removed utilizing a local thermostat in order to keep the temperature constant everywhere in the system. The equilibrium configurations of the system are then sampled according to Boltzmann distribution [500,502,503,505,510,511].

The different resolution of the utilized models typically results in a pressure difference between the corresponding regions which further leads to a non-uniform density profile in the system. Kremer and co-workers [508,512,513] modify the CG potential by introducing a thermodynamic force $f^{th}$ which counterbalances the high pressure of the CG model. This force is obtained in an iterative procedure as

(66) $$f_{i+1}^{th}{\;=\;f}_{i}^{th}\;-\;\frac{\nabla \rho_{i}(r)}{\rho^{*}k_{T}},$$

where $\rho^{*}$ is the reference molecular density, $k_{T}$ is the system’s isothermal compressibility and $\rho_{i}(r)$ is the molecular density profile. This profile is taken as a function of the position in the normal direction to the CG/AA interface. The iterative procedure converges once the density profile is flat, i.e., ∇ρ(r) = 0. The resulting thermodynamic force produces a flat density profile and preserves the thermal compressibility of the system as well as the structure of the system in the CG region. Principally, this method allows one to use any CG force field. As a result, the AA region exchanges energy and molecules with a reservoir like an open system. Such an approach yields a relatively small AA region with the corresponding molecule number fluctuations and all relevant thermodynamic quantities the same as a large AA simulation [508]. It is only because of the thermodynamic driving force that this condition can be achieved independent of the CG model used.

**Figure 17:**
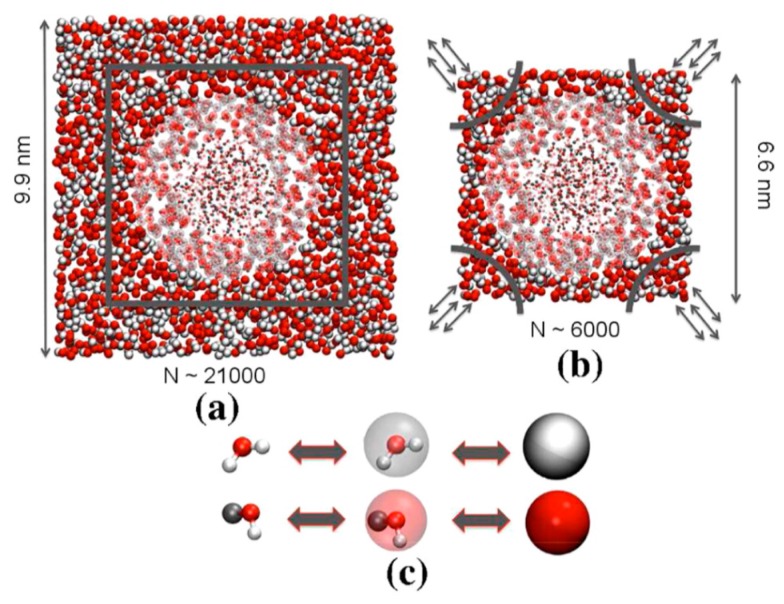
Simulations of a biomolecule dissolved in aqueous methanol: (**a**) Conventional AdResS approach; (**b**) PE-AdResS approach; and (**c**) Mapping scheme of the smooth transition between AA and CG representations. Reprinted with permission from Mukherji and Kremer [518]. Copyright 2016 American Chemical Society.

AdResS provides the possibility to perform simulations of the spatial extension of correlations in the system. Particularly, the structural properties of the AA region can be monitored as a function of its size in order to examine their dependency on the interactions with molecules in the bulk region. For instance, Lambeth et al. [514] used this notion to study the ordering degree of the hydrogen bond network of a molecule with hydrophilic and hydrophobic bonds dissolved in water as a function of the size of the AA region. The extent of spatial correlations in low-temperature para-hydrogen has also been studied with the same approach [515,516]. In some systems, it is critical to have access to a large number of particles, for instance, to precisely evaluate the solvation free energies in mixtures. Thus, a standard AA simulation could lead to extremely costly computations in such cases. Naturally, AdResS has shown to be a viable candidate for these systems as well, as evidenced in some works on methanol-water mixtures [517], and triglycine in aqueous urea [513]. Another interesting possibility for such a case to even further accelerate the simulations was incorporated by Mukherji and Kremer [518] to study a coil-globule transition of a biomolecule in aqueous methanol. In their simulations, the usual closed boundary CG reservoir was replaced with a much smaller open boundary CG reservoir in which particles can be exchanged at the eight corners of the simulation domain, see Figure 17. Through this particle exchange adaptive resolution scheme (PE-AdResS), the depletion effects were avoided during the simulations. This type of *open system* MD simulations have raised attraction in recent years. We refer to the work of Agarwal et al. [519] for instance. Recently, a variation of AdResS formulation was developed by Alekseeva et al. [163] which presents a coupling strategy between the stochastic multiparticle collision dynamics and the deterministic MD methods. In this way, the authors were able to successfully demonstrate that hydrodynamic properties of the mixed fluid are conserved by a suitable coupling of the two particle-based methods.

#### 3.3.2. The Hamiltonian Adaptive Resolution Scheme

A theoretical analysis of the AdResS double-resolution scheme can show that with a local thermostat and the thermodynamic force the atomistic region is equivalent to an open region of a fully atomistic simulation up to second order correlation functions, i.e., the density profile and radial distribution functions [520]. Nonetheless, the lack of a global energy function makes it impossible to perform simulations in the microcanonical ensemble. Consequently, different strategies were employed to formulate an energy conserving version of adaptive resolution simulations including the healing region concept with a space-dependent interpolation of the AA and CG potential energies [521], and the combination schemes for the sum of the Lagrangians of all possible groupings of atomistic and CG molecules [522,523]. Unfortunately, these methods are either inaccurate or extremely complicated to be readily used [337,506]. Recently, an energy-based version of the AdResS method was developed namely the Hamiltonian adaptive resolution scheme (H-AdResS) [524,525]. H-AdResS defines the total Hamiltonian of each molecule with a position-dependent function $H^{tot}$ as

(67) $$H^{tot}{\;=\;K\;+\;U}^{\mathrm{int}}\;+\;\sum_{\alpha }\left\{\psi_{\alpha }U_{\alpha }^{\mathrm{AA}}{\;+\;(1\;-\;\psi }_{\alpha }{)U}_{\alpha }^{\mathrm{CG}}\right\},$$

in which K is the all-atom kinetic energy of the molecules, $U^{\mathrm{int}}$ is the contribution from internal interactions of the molecules, N is the number of molecules, and

(68) $$U_{\alpha }^{\mathrm{AA}}\;=\;\frac{1}{2}\sum_{\beta ,\beta \neq \alpha }^{N}\sum_{ij}U^{\mathrm{AA}}(\left|r_{\alpha i}\;{-\;r}_{\beta j}\right|),$$

(69) $$U_{\alpha }^{\mathrm{CG}}\;=\;\frac{1}{2}\sum_{\beta ,\beta \neq \alpha }^{N}U^{\mathrm{CG}}(\left|R_{\alpha }\;{-\;R}_{\beta }\right|),$$

(70) $$\psi_{\alpha }{\;=\;\psi (R}_{\alpha }).$$

$U_{\alpha }^{\mathrm{AA}}$ and $U_{\alpha }^{\mathrm{CG}}$ represent the potential energies of molecule α in its AA and CG representations, respectively. The force acting on atom i in molecule α can be obtained through differentiation of this Hamiltonian function [337,524,525]. The differentiation operation results in a drift force $F_{\alpha }^{\mathrm{drift}}$ in the transition zone which is proportional to the difference between $U_{\alpha }^{\mathrm{AA}}$ and $U_{\alpha }^{\mathrm{CG}}$, by

(71) $$F_{\alpha }^{\mathrm{drift}}\;=\;-\left[U_{\alpha }^{\mathrm{AA}}\;{-\;U}_{\alpha }^{\mathrm{CG}}\right]\nabla_{\alpha i}\psi_{\alpha }.$$

The definition of the drift force implies that the molecules are pushed into one of the regions if the potentials of the AA and CG regions are different. It is obvious from the mathematical expression of the drift force that it is not possible to write it as a sum of antisymmetric terms with molecule label exchange. Consequently, it results in a local breakdown of Newton’s third law at the transition region. One can deduce that the drift force vanishes if the CG potential perfectly reproduces the many-body potential of mean force in the AA model. Since this is almost never true, a thermodynamic imbalance is always to be expected between the two regions in the form of different pressure and density levels [337,524]. Potestio et al. [524] used a compensation term ${\Delta H(\psi }_{\alpha })$ in the Hamiltonian, as was done in the AdResS method with the thermodynamic force, to correct for this imbalance. The Hamiltonian is therefore modified as [524]

(72) $$\hat{H}=H^{\mathrm{tot}}-\sum_{\alpha =1}^{N}{\Delta H(\psi }_{\alpha }).$$

The authors then obtained an approximate function ${\Delta H(\psi }_{\alpha })$ to cancel out the drift force on average, as

(73) $$\Delta H\left(\psi_{\alpha }\right)\;=\;\frac{{\Delta F(\psi }_{\alpha })\;}{N},$$

in which the suitable compensation term is related to the Kirkwood’s thermodynamic integration for the free energy difference ${\Delta F(\psi }_{\alpha })$ between a hybrid system with a position-independent coupling parameter ($\psi_{\alpha }\;\leq \;1$) and a CG system ($\psi_{\alpha }\;=\;0$) at the reference density $\rho^{*}$ [524]. The authors include a further compensation term to ensure that both the AA and CG subregions coexist at the same reference density $\rho^{*}$ by considering the effect of pressure difference along the interface ${\Delta p(\psi }_{\alpha })$ and re-formulating ${\Delta H(\psi }_{\alpha })$ in terms of the chemical potential gradient $\Delta \mu \left(\psi_{\alpha }\right)$, as [524]

(74) $$\Delta H\left(\psi_{\alpha }\right)\;=\;\Delta \mu \left(\psi_{\alpha }\right)\;=\;\frac{{\Delta F(\psi }_{\alpha })\;}{N}+\frac{{\Delta p(\psi }_{\alpha })}{\rho^{*}}.$$

The H-AdResS method was utilized with both a free energy and a chemical potential compensation strategy to study their effects on the density and pressure profiles [524]. The results showed that with the application of the free energy compensation Equation (73) the pressure profile became flat, but the density was still higher in the AA region. On the other hand, when the chemical potential compensation Equation (74) was applied, the densities of the AA and CG regions attained the same value with a small deviation due to the fluctuations present in the transition region. This was achieved by modifying pressures in each region to correspond to the desirable reference state of density and temperature.

The existence of a Hamiltonian in H-AdResS allows for the precise formulation of a statistical physics theory of double-resolution systems, providing a deep insight into the properties of a given AA model, its CG counterpart and the relation between them. In addition, H-AdResS makes it possible to perform simulation in the microcanonical ensemble as well. Some simulation techniques such as MC can also be incorporated in H-AdResS in contrast to AdResS [525]. It should be noted that H-AdResS along with its compensation strategy can be extended to multicomponent systems. In order to illustrate the routine, a simple case was outlined by Potestio et al. [337] for a liquid composed of two types of molecules.

### 3.4. Extending Atomistic Simulations

Besides the methods that are explicitly designed to link computational techniques from different realms together, there are some approaches to extend the reaches of a specific technique such as MD. As it was noted before, MD plays a critical role in the modelling of materials problems because MD simulations can follow the actual dynamical evolution of the system along its deterministic pathway. However, MD is strictly limited to very short time scales due to its full atomistic representation of the molecules. Therefore, some researchers studied different methods to address the time scale problem including hyperdynamics [526,527,528], parallel replica dynamics [529], and temperature-accelerated dynamics [530]. These methods are based on the transition state theory in which the system trajectory is simulated to find an appropriate pathway to escape from an energy well [528,531]. The simulation walks through this pathway with a process that takes place much faster than the direct MD.

The hyperdynamics is an accelerating approach for MD simulations which needs no prior information about the possible state trajectories of the system in the phase space. The method raises the energy of the system in regions other than at the dividing surfaces of the initial and final configurations in the phase space by applying a bias potential. Consequently, an accelerated transition is achieved from one equilibrium state to another equilibrium state [528]. The parallel replica dynamics method was incorporated for a system with infrequent events in which successive transitions are uncorrelated [529]. In such a system, running a number of independent MD simulations in parallel gives the exact dynamical evolution between the states. For a system with correlated crossing events, the state-to-state transition sequence is still correct. However, the error associated with the simulation time should be eliminated. Finally, in the temperature-accelerated dynamics method, the state-to-state transition is accelerated by increasing the temperature followed by filtering out the transitions that should not have occurred at the original temperature [530]. Consistent with other accelerated dynamics methods, the trajectory of the system is allowed to wander on its own to find an appropriate escape path. Consequently, no prior information is required about the nature of the involved phenomena [528].

The accelerated dynamics methods are formulated in order to find transition pathways between two known equilibrium states via effective MD simulations. Other approaches to extend atomistic simulations are also available which often require no preconceived mechanism or transition state. In order to find the transition pathway, one such method minimizes the average of the potential energy along the path instead of finding the path with the lowest barrier [532,533,534]. Another approach utilizes statistical sampling of the dynamical paths i.e., MC sampling of MD trajectories introducing transition path-sampling methods [535,536,537,538,539]. In addition to these methods, a finite-temperature string method is also available which represents the collection of the hyperplanes normal to the pathways of a system by a string [540,541,542,543]. In this method, the string is constantly updated during the simulations to capture the correct coordinate associated with the phenomenon. Finally, some works try to find dynamical paths that could connect an initial state to a final state in general terms [544,545,546,547,548,549,550]. Such methods often offer good numerical stability, efficient parallelizability, and high quality trajectories.

A class of methods attempts to address the systems with a free-energy surface which could possess several local minima in the free-energy surface. These strategies are generally known as the methods to escape the free-energy local minima [551]. For instance, a proper combination of CG dynamics with the adaptive bias potential methods could allow for the system to avoid local minima in the free-energy surface [551]. At the same time, the system provides a quantitative description of the free-energy surface through the integrated process. Such an approach has especially found application in biological systems [552,553,554].

In a category of systems an inherent dispersity in some characteristic details results in a natural disparity in time scales. A well-known example of such a case was already discussed in Section 2.1, i.e., the Born–Oppenheimer approximation [45], in which the electrons move independently from the nuclei due to their largely different masses. Another scenario which could lead to the separation of time scales is when a subset of forces is much stronger than the rest of the forces, while the masses of the constituents are almost equal. In order to deal more efficiently with such systems, various integration algorithms with multiple time steps have been developed [555]. This idea is particularly useful in polymers in which the bonds vibrate often much faster than they translate and rotate. Consequently, the configuration space as well as the forces can be divided into fast and slow components. As a result of this separation, a set of equations of motion are derived for the development of the fast and slow processes. This set of equations are solved using the multiple-time-step integration in which a small time step Δt to advance the fast processes by n steps while holding the slow variables fixed. The slow processes are then updated using a time step of nΔt. In the case that an analytic solution of high-frequency motions is available, this solution can be incorporated into an integration scheme for the entire system. Therefore, a time step can be defined based on the slow processes and used for the simulation of entire system with a much smaller number of cycles [555].

**Figure 18:**
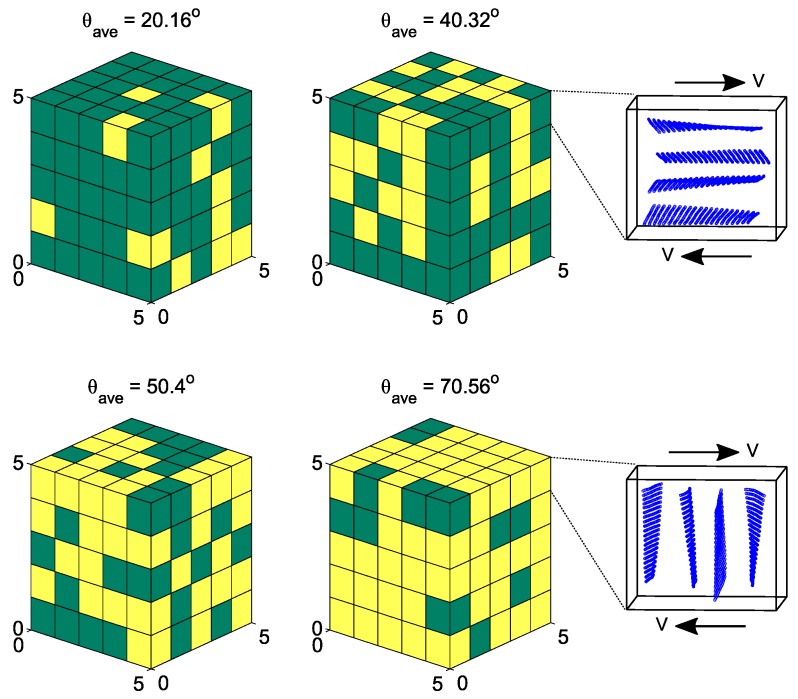
Examples of construction of a large cell for the upper scale simulation benefitting from a random mixing of unit cells resulting in various average initial orientation angles, $\theta_{\mathrm{ave}}$. The initial configurations of the unit cells before the flow starts are also given. Reprinted from Gooneie et al. [196]. Copyright 2016, with permission from John Wiley & Sons Inc.

**Figure 19:**
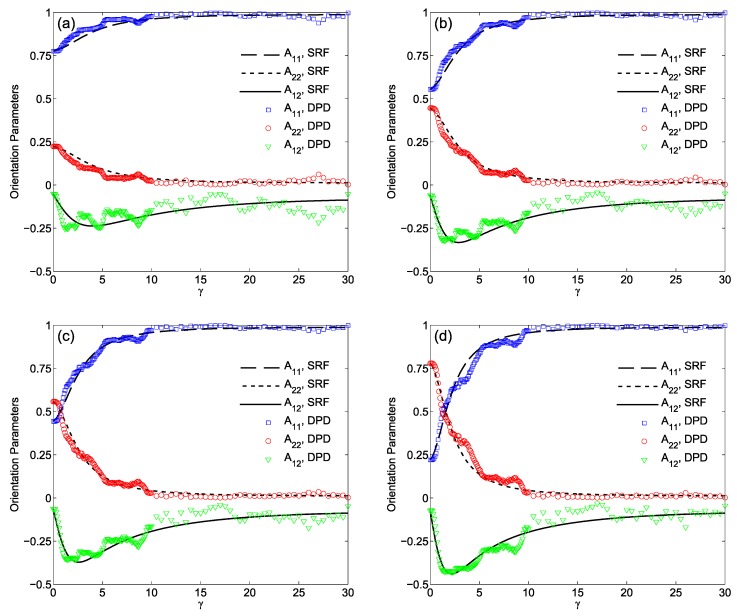
The orientation process defined by the orientation parameters as a function of the shear strain, γ. The results are derived from DPD models and strain reduction factor (SRF) model for various average initial orientation angles of (**a**) $20.16^{{}^{\circ}}$ ; (**b**) $40.32^{{}^{\circ}}$ ; (**c**) $50.40^{{}^{\circ}}$ ; and (**d**) $70.56^{{}^{\circ}}$ . Reprinted from Gooneie et al. [196]. Copyright 2016, with permission from John Wiley & Sons Inc.

In order to extend the time scale of MD simulations, a method was developed based on optimization of the action functional [534]. The method parametrizes the system trajectory as a function of length rather than time. In order to achieve this goal, this approach optimizes an action term defined based on the stochastic time-dependent difference equation rather than solving the Newton equations in MD simulations. A similar idea was recently proposed in which the trajectories of the orientation process of weakly-interacting layered silicates were parametrized as a function of the shear strain instead of the time [196]. The idea of using the applied strain was motivated by the experimental reports supporting strain-dependent structure developments in such non-Brownian materials. Benefitting from the notion that the orientation kinetics is principally determined with respect to strain, the applied strain was selected to pass the orientation parameters to an upper scale through a simple combination of affine and nonaffine deformations, see Figure 18 and Figure 19. This methodology could be also incorporated to develop multiscale models of orientation process provided that the interactions between the components are carefully defined in the unit cell.

## 4. Conclusions and Outlooks

The development of polymeric materials necessitates a comprehensive understanding of the phenomena at different time and length scales. This need has significantly accelerated the progress in theoretical and computational methods to capture the inherent hierarchical phenomena in such materials. In this field, the development of efficient multiscale approaches could lead to the design of materials simultaneously on many scales instead of trial-and-error experimentations. The present review attempted to survey the state-of-the-art of various multiscale simulation approaches as applied to polymer science.

Within the context of an overall multiscale simulation perspective, various approaches for modelling relevant processes in polymer science are classified into three major categories, namely sequential, concurrent, and adaptive resolution approaches. This classification provides the opportunity to easily examine these methods and the systems to which they have been often applied. It is fairly clear from this review that different multiscale approaches provide precious insights into the structure and dynamics of polymeric materials.

In general, the sequential techniques are more popular in polymer science. However, *a priori* knowledge of relevant physical quantities is a prerequisite in these methods. The bridging of various scales in a sequential method is often implicit. A successful sequential modelling depends critically on the accuracy of the finer scale model as well as the reliability of the message-passing algorithms. The link between QM data and atomistic models should be further developed to reproduce the correct structure and thermodynamics. Phenomena which might involve the breaking of bonds require a reactive force field of MD in combination with QM which further complicates the computations as well as the derivation of such a force field from the parametrization of QM data in the first place. Moreover, the construction of CG potentials from atomistic data might necessitate more rigorous strategies particularly in systems with variant local structures and properties. Systematic coarse-graining and backmapping schemes were revisited as major routes towards a sequential model generation in polymers. An inevitable question that arises with the coarse-graining procedure is the question of transferability of the final CG model. As an advantageous aspect, however, the investigation of transferability conditions could help to gain insight into fundamental principles that control the behavior of the system. It is expected that a general prescription for coarse-graining should be developed which ensures a wide range of transferability. In the context of systematic coarse-graining methods, it is interesting to extend super CG models to describe phenomena, such as flow birefringence and systems such as multicomponent mixtures.

The concurrent multiscale methods are a lot more complicated and computationally expensive than sequential approaches particularly when it comes to simulating flow problems. Nevertheless, they do not depend on *a priori* knowledge of relevant physical quantities supplied from smaller-scale simulations. In concurrent methods, it is significant that the problem is carefully posed to make the method practical. The common problem in a concurrent approach is usually associated with the partitioning of domains in the system. More importantly, an appropriate handshaking strategy in a concurrent approach between different domains, which is both mathematically accurate and physically consistent, is challenging and critical. There is no general consensus on what a proper coupling of domains is. Therefore, a general criterion that measures the quality of handshaking between domains would be extremely beneficial. Additionally, there is plenty of room for innovative research on the issue of domain coupling. Although many concurrent approaches exist which are very desirable and appealing in metals and carbon nanomaterials, their use in polymeric systems is still limited to a large extend. In this paper, we have devoted an entire section to cover the fundamentals of several concurrent methods and introduce the existing possibilities to polymer scientists. In order to better illustrate the outlooks, several examples from relevant areas of polymer research are provided so that the reader is persuaded to follow these highlights.

A third group of multiscale simulation strategies was also noted as the adaptive resolution schemes in which a molecule can freely move in space and change its resolution depending on spatial criteria. There is plenty of room in this class of methods for future innovation, either in its methodological aspects or its extension to different materials and phenomena. The method is fundamentally developed for quiescent conditions and the application of flow is yet to be added to these schemes. Even for the simulation of equilibrium conditions, these schemes show noticeable discontinuities in pressure and density profiles at the transition region between the high and low resolutions. Furthermore, the combination of mixed resolution concurrent methods and adaptive resolution schemes can potentially become an increasingly robust multiscale simulation methodology for complex polymer systems. Future work in this area appears to be promising.

When dealing with computer simulations, the role of the computer itself should be also noted including both hardware and software characteristics. Computer technology develops at an astonishing rate. It is believed that the progress in graphics processing units (GPUs) along with the development of GPU-oriented molecular simulation algorithms should extend our reach to yet unexplored spatial and temporal scales in the simulations of polymer systems. Such computational resources along with advanced simulations schemes can closely mimic the problem at hand on engineering time scales in a computer experiment. As a possible area for future endeavors, it would be ideal to compile a combination of atomistic methods with mesoscale and even continuum methods within one simulation package instead of many scattered codes which are available today, each coming with its certain advantages and shortcomings. Such a package could ultimately use the strengths from various individual codes to mitigate for the shortcomings of others. Even more important is the development and implementation of seamless multiscale modelling techniques in this hypothetical package. In addition, it is expected that the qualitative description of fundamental processes will be replaced with the quantitative prediction of material properties with the introduction of exascale computing. First-principle simulations are expected to play an increasing role in these areas. However, the availability of increased computing power will not be sufficient on its own and advanced strategies and techniques are an indispensable part of extreme-scale computing architectures.

Although multiscale methods have brought about substantial developments in the field, the challenge of bridging the time scale of atomic motions to the typical experimental and engineering scales is still far from completion. For instance, in a number of polymer systems such as PNCs, suitable theoretical frameworks are still missing which can provide insights into the nonequilibrium phenomena and the impact of external fields on the morphology and dynamics of the system. Moreover, more rigorous and direct quantitative analysis of nonequilibrium atomistic polymeric models and their CG counterparts is still needed. Various topics still remain to be disclosed in future research including new emerging possibilities to pass the information from the atomic to macroscopic scale and back. Multiscale modelling techniques are yet to be applied to characterize many interesting systems such as polymer flow in dilute and concentrated solutions, characteristics of a polymer layer next to the surface of nanoparticles in PNCs, the molecular roots of the viscoelasticity in filled elastomers, dynamics of confined polymers, etc. These examples are just a few among many topics for the future research on polymer systems. With the progress in theoretical as well as experimental techniques, finding answers to such challenges shall result in a comprehensive knowledge of various material properties of polymeric systems across a range of length and time scales. Moreover, it will bring forth directions to design new systems with desired or yet unexplored properties in the future.

In the framework of multiscale methods, one should not forget that there is also a critical necessity to design new and improved simulation methods at individual time and length scales. From the discussions provided in this review, it is clear that multiscale modelling is a heavily active field in modern science with a multidisciplinary character. The actual power of multiscale strategies is only truly appreciated by overcoming traditional barriers between various scientific disciplines. The computational multiscale approaches should eventually fulfill their philosophy which is to enhance our knowledge of, and ability to control complex processes, even in life sciences. Developing proper multiscale methods is extremely difficult but undeniably represents the future of polymer science as well as computer simulation and modelling.

## Appendix A. Acronyms and Nomenclature

**Acronyms**	
Acronym	Full phrase
AA	All-Atomistic
AC	Amorphous Cell method
AdResS	Adaptive Resolution Scheme
AIMD	Ab Initio Molecular Dynamics
AtC	Atomistic/Continuum method
BD	Brownian Dynamics
BDM	Bridging Domain Method
BGK-LB	Bhatnagar, Gross, And Krook LB method
BSM	Bridging Scale Method
CACM	Composite Grid Atomistic/Continuum Method
CADD	Coupled Atomistic and Discrete Dislocation method
CFD	Computational Fluid Dynamics
CG	Coarse-Grained
CGMD	Coarse-Grained Molecular Dynamics
CLS	Coupling of Length Scales method
CRW	Conditional Reversible Work
D2Q9	2-dimensional lattice with 9 allowed velocities used in LB simulations
D3Q19	3-dimensional lattice with 19 allowed velocities used in LB simulations
DDFT	Dynamic Density Functional Theory
DFT	Density Functional Theory
DPD	Dissipative Particle Dynamics
EFCG	Effective Force CG
EM	Energy Minimization
FDM	Finite Difference Method
FE	Finite Element
FEAt	Finite-Element/Atomistic method
FEM	Finite Element Method
FVM	Finite Volume Method
GDM	Generalized Differences Methods
GFEM	Galerkin Finite Element Method
GPU	Graphics Processing Unit
H-AdResS	Hamiltonian Adaptive Resolution Scheme
HSM	Hybrid Simulation Method
IBI	Iterative Boltzmann Inversion
IMC	Inverse Monte Carlo
LB	Lattice Boltzmann
LGCA	Lattice Gas Cellular Automata
LSM	Lattice Spring Model
MC	Monte Carlo
MD	Molecular Dynamics
Na-MMT	Sodium Montmorillonite
NEMS	Nano-Electro-Mechanical Systems
OpenFOAM	Open Source Field Operation And Manipulation
PA	Polyamide
PAC	Pseudo Amorphous Cell method
Pe	Peclet number
PE	Polyethylene
PNC	Polymer Nanocomposite
PP	Polypropylene
pPMF	Pair Potential of Mean Force
PRISM	Polymer Reference Interaction Site
PS	Polystyrene
PTT	Poly(Trimethylene Terephthalate)
QC	Quasicontinuum method
QM	Quantum Mechanics
QUICK	Quadratic Upstream Interpolation for Convective Kinematics
Re	Reynolds number
RVE	Representative Volume Element
SCFT	Self-Consistent Field Theory
SDPD	Smoothed Dissipative Particle Dynamics
SEM	Spectral Element Method
SPH	Smoothed Particle Hydrodynamics
SRF	Strain Reduction Factor model
SUPG	Streamline-Upwind/Petrov-Galerkin
TB	Tight Binding
TDGL	Time-Dependent Ginzburg-Landau
VMS	Variational Multiscale methods
We	Weissenberg number
XRD	X-Ray Diffraction

**Nomenclature**	
**Symbol**	**Meaning**
A	${A\;=\;6\xi k}_{B}T$ in BD method
$A_{ij}$	maximum repulsion between bead i and bead j in DPD method
$a_{i}$	acceleration of ith particle
$B^{A}$	atomistic domain in concurrent simulations
$B^{C}$	continuum domain in concurrent simulations
$B^{H}$	handshake region in concurrent simulations
$B^{I}$	interfacial region in concurrent simulations
$B^{P}$	padding region in concurrent simulations
$b_{i}$	fitting parameter
$c_{i}$	fitting parameter
$D^{\vartheta }$	the diffusion term of ϑ
$D_{\mathrm{cm}}$	center-of-mass self-diffusion coefficient
e	element
𝕖	absolute unit charge of an electron
$E_{f}$	Young’s modulus
$E_{i}$	energy of atom, particle, or node i
$\bar{E_{i}}$	energy of the ith representative atom in QC method
$E_{k}$	eigenstate of energy
$E_{k_{el}}$	eigenstate energy of an electron
$E_{k_{n}}$	eigenstate energy of a nucleon
$E^{tot}$	total energy
${\Delta F(\psi }_{\alpha })$	free energy difference in H-AdResS method
$F_{ij}^{C}$	conservative force between bead i and its neighboring bead j within the force cutoff radius $r_{cut}$
$F_{ij}^{D}$	dissipative force between bead i and its neighboring bead j within the force cutoff radius $r_{cut}$
$F_{ij}^{R}$	random forces between bead i and its neighboring bead j within the force cutoff radius $r_{cut}$
$F_{\alpha }^{\mathrm{drift}}$	drift force of molecule α
$\bar{f}$	vector of applied forces in the FE region of a concurrent simulation
$f_{i}$	force acting on the ith atom, particle, or node
$f_{\alpha \beta }$	force acting between molecules α and β
$f^{th}$	thermodynamic force
$f_{i}^{B}$	Brownian random force acting on the ith particle
$f_{\alpha \beta }^{\mathrm{AA}}$	atomistic forces acting on molecule α due to the interaction with molecule β
${\;f}_{\alpha \beta }^{\mathrm{CG}}$	CG forces acting on molecule α due to the interaction with molecule β
G′	storage modulus
G″	loss modulus
${H(\Gamma }_{i})$	Hamiltonian of the system at system state $\Gamma_{i}$
$\hat{H}$	modified Hamiltonian of the H-AdResS method
${\Delta H(\Gamma }_{i\to j})$	change in the system Hamiltonian for going from system state $\Gamma_{i}$ to $\Gamma_{j}$
${\Delta H(\psi }_{\alpha })$	compensation term in the Hamiltonian of the H-AdResS method
$H_{\mathrm{FE}}(u_{\alpha },{\dot{u}}_{\alpha })$	Hamiltonian of the FE region as a function of the nodal displacements $u_{\alpha }$, and time rate of nodal displacements ${\dot{u}}_{\alpha }$
$H_{\mathrm{FE}/\mathrm{MD}}(r_{j},v_{j},u_{\alpha },{\dot{u}}_{\alpha })$	Hamiltonian of the FE/MD handshake region as a function of the atomic positions $r_{j}$, atomic velocities $v_{j}$, nodal displacements $u_{\alpha }$, and time rate of nodal displacements ${\dot{u}}_{\alpha }$
$H_{MD}\left(r_{j}{,v}_{j}\right)$	Hamiltonian of the MD region as a function of the atomic positions $r_{j}$, and atomic velocities $v_{j}$
$H_{MD/TB}\left(r_{j}{,v}_{j}\right)$	Hamiltonian of the MD/TB handshake region as a function of the atomic positions $r_{j}$, and atomic velocities $v_{j}$
$H_{TB}\left(r_{j}{,v}_{j}\right)$	Hamiltonian of the TB region as a function of the atomic positions $r_{j}$, and atomic velocities $v_{j}$
$H_{tot}$	total Hamiltonian
h	Planck’s constant
$J^{\vartheta ,\text{C}}$	convection flux term in FVM formulation
$J^{\vartheta ,D}$	diffusion flux term in FVM formulation
K	the all-atom kinetic energy of the molecules
$k_{B}$	Boltzmann’s constant
$k_{T}$	isothermal compressibility
l	bond length
M, $M_{w}$	molecular weight
m	mass of an atom or particle
$m_{el}$	mass of an electron
$m_{n}$	mass of a nucleon
N	number of atoms, particles, or nodes
$N_{c}$	number of monomers per chain
$N_{e}$	number of elements
$N_{q}$	number of quadrature points in the numerical integration
$N_{r}$	number of representative atoms in QC method
P	the projection matrix
${\Delta p(\psi }_{\alpha })$	pressure difference along the interface in H-AdResS method
$p_{i\to j}$	probability of accepting a new configuration for going from system state $\Gamma_{i}$ to $\Gamma_{j}$
$p^{R}$	probability distribution function
$p_{\mathrm{target}}^{R}$	the target probability distribution function of AA simulations
$Q^{\vartheta }$	the generation/destruction of ϑ within the control volume per unit volume
R(u)	residual form of a partial differential equation in terms of the unknown function u in FEM scheme
$R_{g}$	radius of gyration
$R_{i}$	center of mass coordinates of the ith molecule
r	coordinates vector of an atom, or particle, or node
r	distance
$r_{cut}$	force cutoff radius
$r_{{el}_{i}}$	spatial coordinates of an electron
${\hat{r}}_{ij}$	unit vector pointing from the center of bead j to that of bead i
$r_{n_{j}}$	spatial coordinates of a nucleon
$r_{e}^{\mathrm{cent}}$	coordinates of the Gauss point in element e taken at the centroid of the triangular elements
$r_{e}^{q}$	position of quadrature point q of element e in the reference configuration
${\delta r}_{i}^{B}(t+\Delta t)$	random displacement of the ith particle due to the random forces during time step Δt
S	surface vector
$S_{i}$	ith subregion
$\left\{S_{\varrho }\right\}$	set of weighting functions in FEM
$s_{\mathrm{entropy}}$	rescaling factor for the entropy change
$s_{\mathrm{friction}}$	rescaling factor for the friction change
T	temperature
t	time
∆t	time step
U(r)	potential energy
$U^{A}$	potential energies of the atomistic region
$U^{\mathrm{atom}}$	energy functional of a systems assuming it is entirely modelled using atoms
$U^{C}$	potential energies of the continuum region
$U^{\mathrm{CG}}\left(r,l,\theta ,℧\right)$	general form of the CG potential function in IBI method
$U^{\mathrm{FE}}$	energy functional of a systems assuming it is entirely modelled using FEM
$U^{H}$	potential energies of the handshake region
$U^{\mathrm{int}}$	energy of internal interactions
$U^{tot}$	total potential energy of the entire system
$U_{\mathrm{angle}\;}^{\mathrm{CG}}(\theta )$	bond angle potential in the blob model
$U_{\mathrm{bond}\;}^{\mathrm{CG}}(l)$	bond potential in the blob model
$U_{\mathrm{nonbonded}\;}^{\mathrm{CG}}(r)$	potential of nonbonded interactions in the blob model
$U_{\alpha }^{\mathrm{AA}}$	potential energy of molecule α in the AA representation
$U_{\alpha }^{\mathrm{CG}}$	potential energy of molecule α in the CG representation
u	vector of nodal displacements in the FE region of a concurrent simulation
u(r)	the unknown function in FEM which one needs to find
$u_{h}(r)$	approximation of the function u(r) under consideration in FEM
$u_{\alpha }$	displacements of atom, particle, or node α
${\dot{u}}_{\alpha }$	rate of displacements of atom, particle, or node α
$u^{n}$	values of the function $u_{h}$ at node n of the mesh
$V_{e}$	volume of element e
dV	volume element of the simulation domain in FEM
$\partial V_{e}$	surfaces surrounding the volume $v_{e}$ of element e
v	macroscopic velocity magnitude
𝕧	$𝕧\;=\;\sqrt{3\;}{𝕧}_{s}$ in LB method
v(r,t)	macroscopic local velocity at node r at time t in LB
$\tilde{v}(t+∆t)$	estimated velocity in the next time step using a predictor method in DPD velocity-Verlet algorithm
${\delta v}_{i}^{B}(t+\Delta t)$	Random velocity change of the ith particle due to the random forces during time step Δt
$v_{i}$	velocity of ith atom, particle, or node
$\left|{𝕧}_{i}\right|$	velocity magnitude in i-direction in LB method
$\left\{{𝕧}_{k}\right\}$	set of prescribed velocity vectors connecting the neighboring nodes in LB method
${𝕧}_{s}$	speed of sound
W	a function of deformation gradient Δ
$w_{i}$	weighting constants used in LB method
$z_{n}𝕖$	positive unit charge of a nucleon
$\Gamma_{i}$	system state in a phase space at position i
γ	exact solution in the projection method
$\dot{\gamma }$	shear-rate
$\bar{\gamma }(r_{\alpha })$	coarse scale solution of a problem in the projection method
γ′	fine scale solution of a problem in the projection method
Δ	deformation gradient
δ	delta function
$∆\mu \left(\psi_{\alpha }\right)$	chemical potential gradient in H-AdResS method
ε	neighboring cells of a specific element in FVM
ζ	random number between 0 and 1 which is to determine the acceptance or rejection of a new configuration
$\zeta_{ij}$	a Gaussian random number with zero mean and unit variance used in the definition of the random forces between beads i and j in DPD method
η	viscosity
Θ	a weighting function to link FE and atomistic models in concurrent simulations
θ	bond angle
$\theta_{\mathrm{ave}}$	averaged initial orientation angle
$\Lambda_{ik}$	collision matrix used in LB method
λ	multiplication parameter in in DPD velocity-Verlet algorithm
μ	fitting parameter
ν	fitting parameter
ϑ	a general conserved scalar variable in FVM scheme
ξ	friction coefficient between atoms or particles
$\xi_{ij}$	friction coefficient between bead i and bead j in DPD method
$\xi_{m}$	friction coefficient between particles of freely-rotating chains
ϖ	wave function of electrons
ρ	fluid density in CFD
ρ(r,t)	macroscopic local density at node r at time t in LB method
$\rho_{i}(r)$	molecular density profile in the ith iteration step as a function of the position in the direction perpendicular to the interface, in AdResS method
$\rho^{*}$	reference molecular density
$\varrho_{i}$	ith weighting function in FEM
$\sigma_{ij}$	noise amplitude between bead i and bead j in DPD method
$\varsigma_{i}^{\alpha }$	shape function of node i evaluated at the point with coordinates $r_{\alpha }$
τ	characteristic collision time in LB method
Φ(u)	integral form of the weighted residuals in FEM
${\varphi (r)}_{k}$	wave function in Schrödinger’s equation
ϕ	wave function of the nuclei
$\chi_{ij}$	a parameter in DPD formulation which equals 1 for beads with a distance less than $r_{cut}$ and equals 0 otherwise
$\Psi_{i}(r,t)$	particle distribution function used in LB at node r at time t moving with velocity ${𝕧}_{i}$ In the i-direction
$\Psi_{i}^{\mathrm{eq}}(r,t)$	equilibrium particle distribution function used in LB at node r at time t moving with velocity ${𝕧}_{i}$ In the i-direction
ψ	spatial interpolation function in AdResS method
$\psi_{n}(r)$	interpolation functions in FEM for node n
$\psi_{n}^{e}\left(r\right)$	interpolation functions in FEM for node n in element e
Ω	simulation domain in FEM
∂Ω	boundaries of the simulation domain in FEM
℧	dihedral angle
ω	Frequency
$\omega_{i}$	quadrature weight signifying how many atoms a given representative atom stands for in the description of the total energy, in QC method
$\omega^{D}\left(r_{ij}\right)$	dissipative weight function in DPD method
$\omega^{q}$	associated Gauss quadrature weights of quadrature point q of element e
$\omega^{R}\left(r_{ij}\right)$	random weight function in DPD method
